# Evaluation of thermodynamics, formation energetics and electronic properties of vacancy defects in CaZrO_3_

**DOI:** 10.1038/s41598-017-08189-2

**Published:** 2017-08-16

**Authors:** Syed Muhammad Alay-e-Abbas, Safdar Nazir, Stefaan Cottenier, Ali Shaukat

**Affiliations:** 1 Department of Physics, Government College University Faisalabad, Allama Iqbal Road, 38000 Faisalabad, Pakistan; 2 Department of Physics, University of Sargodha, 40100 Sargodha, Pakistan; 3 Center for Molecular Modeling, Ghent University, Tech Lane Ghent Science Park – Campus A, building 903, 9052 Zwijnaarde, Belgium; 4 Department of Electrical Energy, Metals, Mechanical Constructions and Systems, Ghent University, Tech Lane Ghent Science Park – Campus A, building 903, 9052 Zwijnaarde, Belgium; 5 Department of Physics, The University of Lahore, Sargodha Campus, Sargodha, Pakistan

## Abstract

Using first-principles total energy calculations we have evaluated the thermodynamics and the electronic properties of intrinsic vacancy defects in orthorhombic CaZrO_3_. Charge density calculations and the atoms-in-molecules concept are used to elucidate the changes in electronic properties of CaZrO_3_ upon the introduction of vacancy defects. We explore the chemical stability and defect formation energies of charge-neutral as well as of charged intrinsic vacancies under various synthesis conditions and also present full and partial Schottky reaction energies. The calculated electronic properties indicate that hole-doped state can be achieved in charge neutral Ca vacancy containing CaZrO_3_ under oxidation condition, while reduction condition allows to control the electrical conductivity of CaZrO_3_ depending on the charge state and concentration of oxygen vacancies. The clustering of neutral oxygen vacancies in CaZrO_3_ is examined as well. This provides useful information for tailoring the electronic properties of this material. We show that intentional incorporation of various forms of intrinsic vacancy defects in CaZrO_3_ allows to considerably modify its electronic properties, making this material suitable for a wide range of applications.

## Introduction

The alkaline-earth metal zirconate ceramic materials (AZrO_3_, where A: Ca, Sr and Ba) are a versatile class of solid materials which have attracted a renewed research interest owing to their potential utilization in electrical, electronic and optical devices^1–3^. Calcium zirconate, CaZrO_3_ (CZO), is one such ceramic material that crystallizes in the perovskite-like structure and has shown a wide range of device applications^4^. The anisotropic large dielectric tensor of pristine CZO^5^ makes it suitable as a gate material and dielectric resonator for electronic industry^6, 7^. On the other hand, the high chemical stability with silicon, good mechanical strength^8^ and small thermal expansion coefficient^9^ are among salient features of CZO which allow its use in oxygen, hydrogen, and water monitoring applications^10–12^.

The presence of vacancies and impurities in wide band-gap materials can significantly alter their physical properties. In some cases defects in solids may deteriorate useful properties, however these defects sometime lead to systems with physical properties which are promising for certain functional applications^13^. In case of CZO, for instance, the introduction of a Ti dopant gives a linear dielectric system with high energy density and excellent temperature stability, which are prerequisites for materials to be used in electrical power modules operating above 200 °C^14^. On the other hand, a variety of photoluminescence (PL) emission spectra can be achieved in CZO by incorporating aliovalent dopants such as Eu^3+^ ^15^, Tb^3+^ ^16^, and Li^1+^ ^17^, while the presence of carrier traps in un-doped CZO can make it a promising electron transport material (ETM) for optical storage^18^. Doped CZO or CZO containing intrinsic vacancy defects are also promising candidates for achieving proton conductivity under hydrogenated or wet atmosphere at high temperatures^19^. To this end, In_2_O_3_-doped CZO has been thoroughly investigated owing to its higher mechanical and chemical stability and a sufficiently high degree of proton conductivity at elevated temperatures. In fact, In_2_O_3_-doped CZO has been practically used as an electrolyte of galvanic cell-type hydrogen sensor for molten metals^20–22^. Similarly, several reports suggest that disturbing the stoichiometric of CZO can bring about novel mixed *p*-type and ionic conduction behavior that can be controlled by varying the synthesis conditions^23–26^.

Although a lot of experimental work has been undertaken for studying a wide range of properties of CZO, fewer theoretical studies of calcium zirconate are available in literature. First-principles calculations for investigating the structural, mechanical, and electronic properties of cubic and orthorhombic phases of CZO have been carried out by Hou^27^ and Stoch *et al*.^28^, while Brik *et al*.^29^ studied the (001) surfaces of CZO for exploring their electronic properties and energetic stability. On the other hand, the potential for using Yb^3+^-, Nd^3+^-, In^3+^-, Ga^3+^- and Sc^3+^-doped CZO for protonic conduction has lead many researchers to investigate the mechanisms of doping and trapping of H^+^ ion in CZO using quantum mechanical techniques^30–32^. Since the intentional incorporation of intrinsic vacancy defects in doped transition metal perovskite oxides promises profound enhancement in their ionic conductivity^30^, ^33–35^, it is imperative to have a thorough theoretical insight in to the chemistry of intrinsic vacancy defects containing un-doped CZO under various growth conditions^3, 36, 37^. The motivation for present study is further strengthened by the fact that tuning the electronic transport properties of CZO by manipulating vacancy defects has not been carried out so far. To this end, we employ the full-potential linearized augmented plane-wave (FP-LAPW) method within the framework of DFT for investigating the influence of vacancy defect on the electronic structure of CZO. It is expected that the detailed study of vacancy defects and clustering of charge neutral O vacancies reported in this work may stimulate future experimental studies to identify and use non-stoichiometric CZO for advanced device applications.

## Method of Calculation

To evaluate the relative stability of ${V}_{Ca}^{q}$, ${V}_{Zr}^{q}\,$and ${V}_{O}^{q}\,$vacancies (where *q* represents the charge state) in CZO and their corresponding electronic properties, we employ the all-electron FP-LAPW method as implemented in the WIEN2k code^38^. Throughout the whole of this study the exchange-correlation functional is modeled by the Perdew, Burke and Ernzerhof (PBE)^39^ generalized gradient approximation (GGA) parametrization scheme. The FP-LAPW method requires partitioning the crystal by non-overlapping muffin-tin spheres. These spheres are centered at the calcium, zirconium and oxygen sites and are given radii (*R*_*MT*_) of 2.11, 1.99 and 1.80 (in *a.u*.), respectively. FP-LAPW basis functions are constructed from spherical harmonics inside the muffin tin spheres, connected to plane waves in the interstitial region in between. The size of the basis set is controlled by the plane-wave cut-off (*K*_max_). In order to pinpoint a basis set size that ensures sufficient precision, we exploit the fact that a determination of atomic chemical potentials for formation energy calculations depends entirely on the precise computation of enthalpies of formation. Since enthalpies of formation are calculated as the difference of total energies of a compound and its constituent atoms in their standard reference states, we have tested the precision of our results by comparing the enthalpies of formation computed using different choices for the *K*_max_ and for the **k**-mesh used for numerical integration. Our results indicate that using *K*_max_ = 4.444 and 6 × 4 × 6/4 × 4 × 4/12 × 12 × 12 **k**-meshes for CZO/ZrO_2_/CaO the precision in the calculated enthalpies of formation is ±1 meV/atom. Therefore, the total energy calculations of different sizes of supercells used in the present work are performed using *R*_*o*_ × *K*_max_ = 8 (where *R*_*o*_ is the *R*_*MT*_ of O atom), while the maximum values of angular momentum of partial waves inside the muffin-tin spheres, *l*_max_, and the magnitude of vector for Fourier expansion of charge density, *G*_max_, are set at 10 and 18, respectively. As shown in ref. 40, all modern DFT codes make essentially identical predictions for properties that depend on total energies. The conclusions obtained in this work are therefore not affected by our choice for the FP-LAPW method.

**Figure 1:**
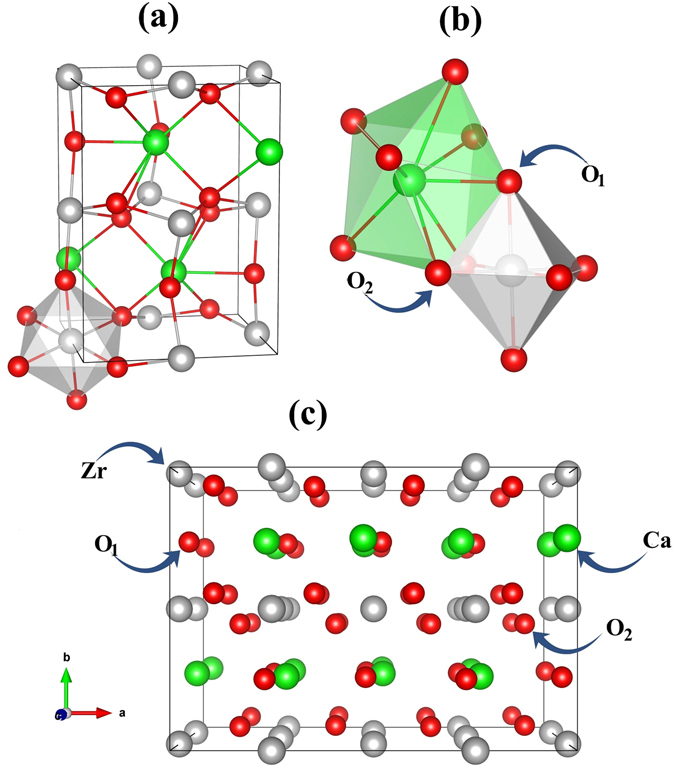
The (**a**) orthorhombic unit cell of CaZrO_3_ along with (**b**) the anion coordination of Ca and Zr with apical (O_1_) and equatorial (O_2_) oxygen atoms. In (**c**) the pristine 2 × 1 × 2 supercell of CaZrO_3_ is shown which was used for constructing supercells containing intrinsic vacancy defects and oxygen vacancy clustering as explained in the text.

CZO crystallizes experimentally in an orthorhombically distorted perovskite structure (space group # 62, *Pnma*) with 20 atoms and lattice parameters *a*_*exp*._ = 5.594 Å, *b*_*exp*._ = 8.021 Å and *c*_*exp*._ = 5.761 Å^41^, as shown in Fig. 1. This experimental unit cell has been used as the starting point for a complete DFT-GGA optimization of unit cell volume, *c/a* and *b/a* ratios and internal geometry using a 6 × 4 × 6 **k**-mesh. The GGA optimized cell is subsequently used for a 2 × 1 × 2 supercell with composition Ca_16_Zr_16_O_48_ (80-atoms), which is sufficiently large for formation energy calculations and electronic properties of defected CZO^42^. A 3 × 4 × 3 **k**-mesh was used for this supercell and the self-consistent procedure to find the ground state density and the self consistent cycle was terminated when the difference in calculated energies between subsequent iterations was less than 10^−5^ *Ry*. For all the defective supercells it was ensured that the atomic positions are fully relaxed, indicated by forces on each atom being below 1 *mRy/a.u*. The k-meshes for vacancy containing supercells of CZO adopting different symmetry structures and the basis set sizes for elemental solids are chosen with reference to the values of k-mesh and *K*_max_ used for performing calculations for bulk unit cell of CZO^38^. The effect of spin-orbit coupling was neglected, after having tested it gave negligible effects on the energetic and electronic properties.

In case of non-stoichiometric CZO containing isolated ${V}_{Ca}^{q}$, ${V}_{Zr}^{q}\,$and ${V}_{O}^{q}\,$vacancies, we construct 2 × 1 × 2 supercells having compositions Ca_15_Zr_16_O_48_, Ca_16_Zr_15_O_48_ or Ca_16_Zr_16_O_47_ that correspond to 6.250%, 6.250% and 2.083% vacancy concentrations, respectively. The oxygen vacancy clustering, on the other hand, is simulated by systematically vacating oxygen sites in the Ca_8_O_8_ and Zr_8_O_16_ layers of the 2 × 1 × 2 supercell (Fig. 1(c)) of CZO for achieving 4.167, 8.333%, 16.667% and 33.333% oxygen vacancy concentration. In case of 4.167% oxygen vacancy concentration the structural models are constructed by removing two distant apical (O_1_) and equatorial (O_2_) oxygen atoms from the Ca_8_O_8_ layer (designated as ${V}_{2{O}_{1}}$) and Zr_8_O_16_ (designated as ${V}_{2{O}_{2}}$) layers of the 2 × 1 × 2 supercell, respectively. On the other hand, 8.333% concentration of ${V}_{O}^{0}\,$is achieved by removing four O atoms from Ca_8_O_8_ (designated as ${V}_{4{O}_{1}}$) and Zr_8_O_16_ (designated as ${V}_{4{O}_{2}}$) layers of the 2 × 1 × 2 supercell. Similar procedure is adopted for obtaining 16.667% oxygen vacancy concentration in the Ca_8_O_8_ (designated as ${V}_{8{O}_{1}}$) and Zr_8_O_16_ (designated as ${V}_{8{O}_{2}}$) layers of the 2 × 1 × 2 supercells (cif files for the optimized geometries of all these supercells are added in the supplementary information). For all the O vacancy clustering cases discussed above, only charge neutral O-vacancies are considered since the balanced charge formula of calcium zirconate (Ca^2+^Zr^4+^(O_3_)^2−^) suggests that an occupied defect energy level can only arise close to the edge of conduction band minimum (CBM) of CZO in case of a charge neutral O vacancy. Furthermore, the ${V}_{O}^{0}\,$clustering cases considered in this work do not lead to spontaneous polarization of defective CZO supercells caused by background charges which are essential for simulating charged vacancy defects^43, 44^.

## Results and Discussion

### Stability Diagram

**Figure 2:**
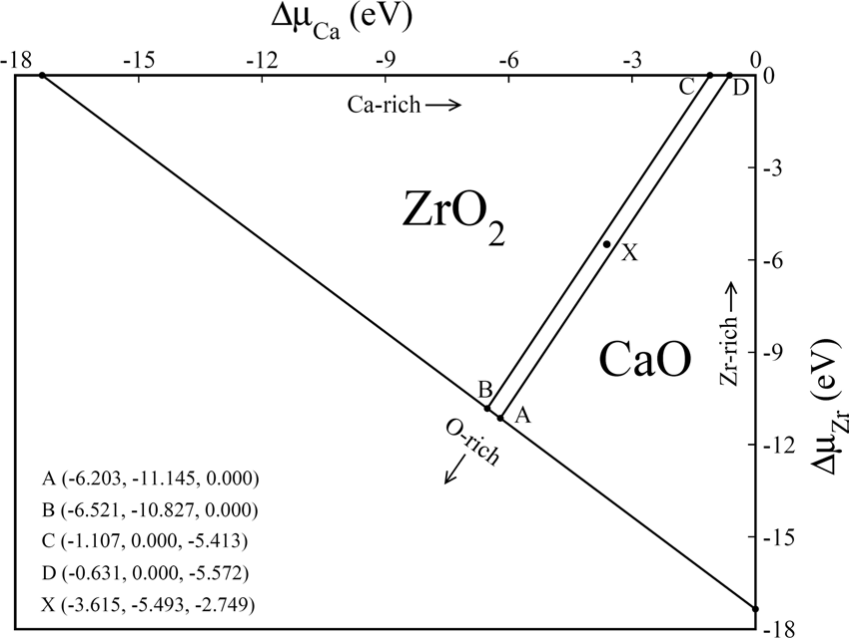
The calculated chemical stability diagram showing the area within which (Δμ_Ca_, Δμ_Zr_, Δμ_O_) coordinates represent stable growth of CaZrO_3_.

For studying the chemical stability of pristine CZO, we have computed the limits of atomic chemical potentials (*Δ*_*μX*_ ≤ 0) by assuming that the chemical potential (*μ*_*X*_) of an isolated atom of species *X* is less than or equal to the chemical potential of that atomic species in its stable solid/gaseous state (${\mu }_{X}^{solid/gas}$). The determination of valid limits of *Δ*_*μX*_ is necessary for computing stability ranges shown in Fig. 2 and enables one to compute formation energies of vacancy defects under different growth conditions^45^. For stable production of CZO without the presence of secondary phases like CaO and ZrO_2_, we vary the values of Δμ_Ca_, Δμ_Zr_ and Δμ_O_ by satisfying the following equations^46^.

1 $$\Delta {H}_{f}^{CaZr{O}_{3}}=\Delta {\mu }_{Ca}+\Delta {\mu }_{Zr}+3\Delta {\mu }_{O}$$

2 $$\Delta {H}_{f}^{CaO}\ge \Delta {\mu }_{Ca}+\Delta {\mu }_{O}$$

3 $$\Delta {H}_{f}^{Zr{O}_{2}}\ge \Delta {\mu }_{Zr}+2\Delta {\mu }_{O}$$

Here $\Delta {H}_{f}^{CaZr{O}_{3}}$, $\Delta {H}_{f}^{CaO}\,$and $\Delta {H}_{f}^{Zr{O}_{2}}\,$are the enthalpies of formation of CZO, CaO and ZrO_2_, respectively, which are computed using

4 $${\rm{\Delta }}{H}_{f}^{CaZr{O}_{3}}={E}^{CaZr{O}_{3}}-{E}^{C{a}_{fcc}}-{E}^{Z{r}_{hcp}}-3\frac{1}{2}{E}^{{O}_{2}}$$

5 $${\rm{\Delta }}{H}_{f}^{CaO}={E}^{CaO}-{E}^{C{a}_{fcc}}-\frac{1}{2}{E}^{{O}_{2}}$$

6 $${\rm{\Delta }}{H}_{f}^{Zr{O}_{2}}={E}^{Zr{O}_{2}}-{E}^{Z{r}_{hcp}}-2\frac{1}{2}{E}^{{O}_{2}}$$

The Gibbs free enthalpy, *G*, of crystalline solid can be distinguished from its total energy, *E*, through the term *−TS* + *pV*. Since the DFT calculations are performed at *T* = 0 and *p* = 0, the formation energy of a solid (e.g $\Delta {E}_{f}^{CaZr{O}_{3}}$) coincides with its enthalpy of formation (e.g $\Delta {H}_{f}^{CaZr{O}_{3}}$). In above equations, *E*’s are the calculated minimum total energies of GGA optimized crystal structures of orthorhombic calcium zirconate, cubic calcium oxide, monoclinic zirconia, face centered cubic (fcc) calcium and hexagonal close packed (hcp) zirconium. The total energy of an *O*_2_ molecule, on the other hand, has been computed by adding the GGA calculated cohesive energy of oxygen^47^ to twice the total energy of a single oxygen atom inside a sufficiently large unit cell using only Γ-point sampling. For the sake of analyzing the error bars in the present DFT calculations, the cohesive energies, *E*_*c*_, of Ca and Zr are also computed. The calculated bare PBE equilibrium volume per atom (*V*_*PBE-bare*_), zero point corrected equilibrium volume per atom (*V*_*corr*._), lattice parameters corresponding to corrected equilibrium volume (*a*_*corr*._, *b*_*corr*._ and *c*_*corr*._), *E*_*c*_ and *ΔH*_*f*_ are presented in Table 1. Except for the case of fcc calcium, one can easily notice that all *V*_*PBE-bare*_ values are overestimated, while the calculated PBE *E*_*c*_ and *ΔH*_*f*_ are underestimated. For the stable crystal structures of the compounds and elemental solids under study, *V*_*corr*._ have been computed by considering a systematic GGA deviation of 3.6% in the calculated volume and using zero-point correction^48^, *ΔV*, of 0.010 Å^3^/atom, 0.016 Å^3^/atom, 0.006 Å^3^/atom, 0.032 Å^3^/atom and 0.007 Å^3^/atom for CZO, CaO, ZrO_2_, fcc Ca and hcp Zr, respectively. Compared to experimental data, all the *V*_*corr*._ and the cohesive energies reside well within the intrinsic error bars proposed by K. Lejaeghere *et al*.^49^. It can also be seen that $|{\rm{\Delta }}{H}_{f}^{Zr{O}_{2}}+{\rm{\Delta }}{H}_{f}^{CaO}| < |{\rm{\Delta }}{H}_{f}^{CaZr{O}_{3}}|$ satisfies the fundamental criteria for the stable production of CZO^50^.

**Table 1:** The calculated *V*_*PBE-bare*_: bare PBE equilibrium volume per atom (Å^3^/atom); *V*_*corr*._: zero-point corrected equilibrium volume per atom (Å^3^/atom); *a*_*corr*._, *b*_*corr*._ and *c*_*corr*._: lattice parameters corresponding to *V*_*corr*_.

	This work	Experimental data
Ca_fcc_		
*V*_*PBE-bare*_	42.831	—
*V*_*corr*._	41.321 ± 1.1	42.877^69^
*a*_*corr*._	5.488 ± 0.117	5.556^69^
*E*_*c*_	−1.880 ± 0.311	−1.843^70^
Zr_hcp_		
*V*_*PBE-bare*_	23.520	—
*V*_*corr*._	22.681 ± 1.1	23.281^71^
*a*_*corr*_	3.197 ± 0.107	3.232^71^
*c*_*corr*_	5.125 ± 0.107	5.147^71^
*E*_*c*_	−6.279 ± 0.311	−6.320^70^
CaO		
*V*_*PBE-bare*_	14.181	—
*V*_*corr*_	13.686 ± 1.1	13.712^72^
*a*_*corr*_	4.784 ± 0.135	4.787^72^
*ΔH*_*f*_	−6.203	−6.582^70^
ZrO_2_		
*V*_*PBE-bare*_	12.029	—
*V*_*corr*._	11.603 ± 1.1	11.720^73^
*a*_*corr*._	5.129 ± 0.191	5.151^73^
*b*_*corr*._	5.181 ± 0.191	5.203^73^
*c*_*corr*._	5.315 ± 0.191	5.316^73^
*β*	99.656	99.200^73^
*ΔH*_*f*_	−10.827	−11.41^74^
CaZrO_3_		
*V*_*PBE-bare*_	13.173	—
*V*_*corr*_	12.709 ± 1.1	12.925^41^
*a*_*corr*_	5.738 ± 0.373	5.594^41^
*b*_*corr*_	7.977 ± 0.373	8.021^41^
*c*_*corr*_	5.553 ± 0.373	5.761^41^
*ΔH*_*f*_	−17.347	−18.281^75^

(Å^3^/atom); *E*_*c*_: cohesive energies (eV/atom); and *ΔH*_*f*_: enthalpy of formation (eV/formula unit); along with experimental data. The residual error bar for zero point corrected equilibrium volume per atom and cohesive energies are explained in the text.

The chemical stability diagram (CSD) of CZO (Fig. 2) has been obtained from the calculated values of *ΔH*_*f*_ presented in Table 1 and by satisfying Equations 1–3. Stable production of CZO can be achieved for all points lying inside the quadrangle ABCD and on the lines joining points A (−6.203, −11.145, 0.000), B (−6.521, −10.827, 0.000), C (−1.107, 0.000, −5.413) and D (−0.631, 0.000, −5.572) and agrees well with CSD that can be computed using experimental *ΔH*_*f*_ values. For example, the calculated (−6.203, −11.145, 0.000) and experimental (−6.582, −11.698, 0.000) chemical potential coordinates at O-rich condition (point A) show good agreement. The calculated chemical potential coordinates shown in Fig. 2 allow us to identify the extreme oxidation condition (O-rich) and the extreme reduction condition (O-poor). Hence, oxidation condition is realized at both point A (Zr-poor condition) and B (Ca-poor condition) where *Δμ*_*o*_ = 0 eV. Moreover, the range of *Δμ*_*o*_from 0 eV to −5.572 eV gives us a wide variety of *Δμ*_*Ca*_and *Δμ*_*Zr*_ values. The metal-rich conditions are true for both point C and D. The point X (−3.615, −5.493, −2.749) is in middle of the stability region for CZO and allows evaluation of intrinsic vacancy defects in CZO under optimal growth conditions.

### Pristine and Neutral Vacancy Defects Containing CaZrO_3_

In the ideal cubic perovskite structure the oxygen atoms form an octahedral and cuboctocahedral coordination with Zr and Ca atoms, respectively, with Zr–O and Ca–O having constant values of bond lengths throughout the cubic unit cell. Under ambient conditions the orthorhombically distorted perovskite-like structure of CZO is found to be stable, which persists up to 1900 °C^28^. As evident from the calculated equilibrium unit cell of CZO shown in Fig. 1(a), the ZrO_6_ octahedra are tilted in the ac-plane, which results in a deviation of both bond length and Zr–O–Zr bond angle from their ideal values. Table 2 provides a comparison between the atomic positions, bond lengths and bond angles computed in this study, and results from previous experimental and theoretical work. In the GGA optimized unit cell of orthorhombic CZO we found the Zr–O–Zr bond angle to be 146.290° and 144.632° with the equatorial (O_2_ atoms residing in the ZrO_2_ layer) and the apical (O_1_ atoms residing in the CaO layer) oxygen atoms, respectively. Due to this tilt of the ZrO_6_ octahedra the  ideal 12 fold coordination of Ca with O in cubic perovskite structure is reduced to an 8 fold coordination in the orthorhombic structure, as shown in Fig. 1(b). The Ca atom is bonded with 8 oxygen atoms having bond lengths that range from 2.345 Å to 2.882 Å. On the other hand, the bond lengths of the Zr atom with its octahedrally coordinated oxygen atoms range from 2.108 Å to 2.118 Å.

**Table 2:** Comparison of the calculated and experimental atomic positions, mean bond lengths (Å), and bond angles (degrees) of pristine orthorhombic (space group # 62,$Pbnm$) CaZrO_3_.

		This work	Previous study^31^	Experiment^42^
Ca	*x*	0.0499	0.0496	0.0479
	*y*	0.2500	0.2500	0.2500
	*z*	0.0132	0.0121	0.0125
Zr	*x*	0.5000	0.5000	0.5000
	*y*	0.0000	0.0000	0.0000
	*z*	0.0000	0.0000	0.0000
O_1_	*x*	−0.0407	−0.0381	−0.0423
	*y*	0.2500	0.2500	0.2500
	*z*	0.6065	0.6032	0.6130
O_2_	*x*	0.2992	0.3007	0.2999
	*y*	0.0566	0.0548	0.0566
	*z*	0.3016	0.3026	0.3011
Zr–O	(x 6)	2.1130	2.096	2.105^76^
Ca–O	(x 4)	2.392	2.382	2.374^76^
Ca–O	(x 4)	2.779	2.762	2.768^76^
Zr–O_1_–Zr		144.632	145.760	143.460^76^
Zr–O_2_–Zr		146.290	146.500	146.350^76^

**Figure 3:**
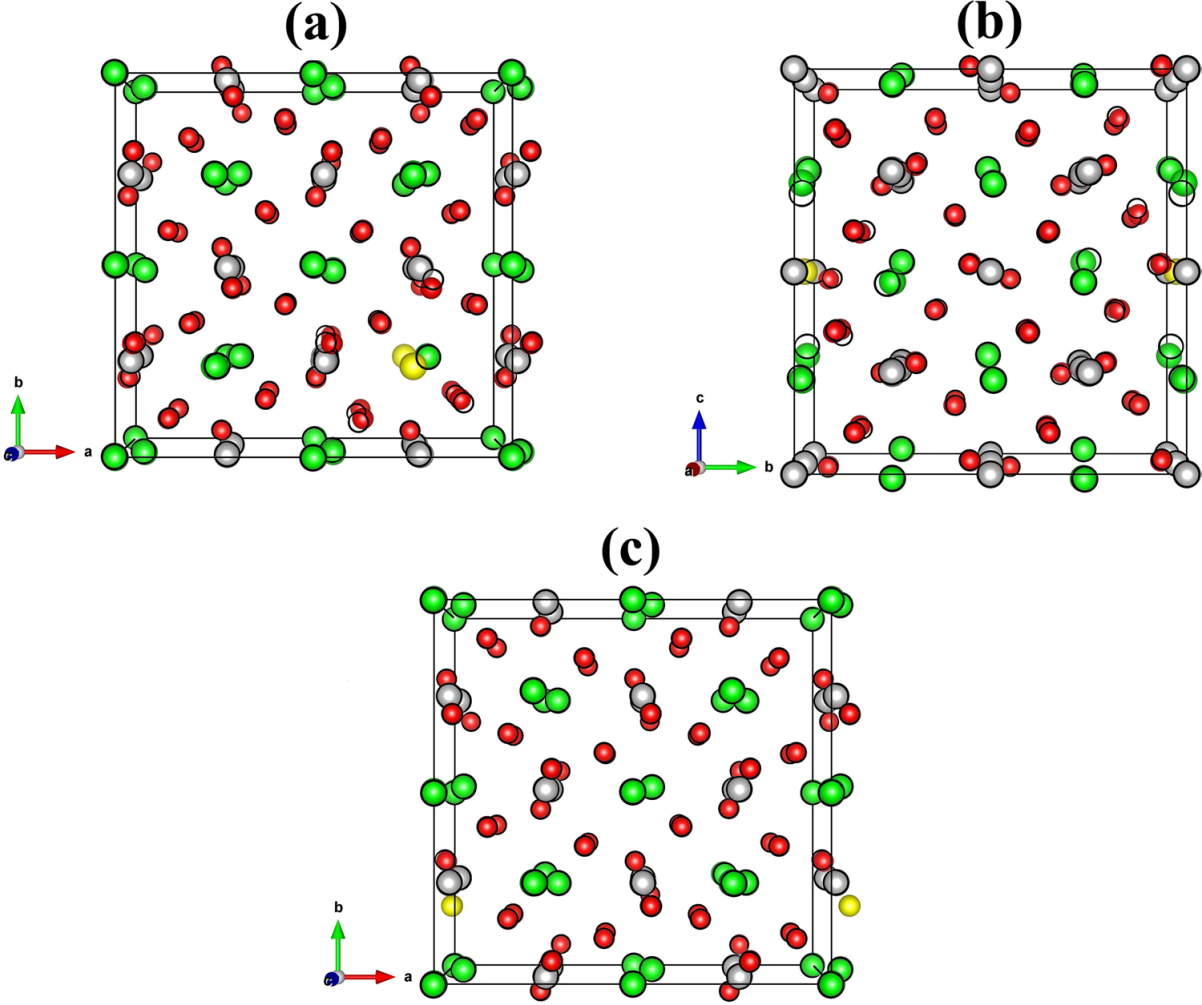
Changes in the positions of atoms before and after the minimization of atomic forces in defective supercells of CaZrO_3_ containing isolated (**a**) ${V}_{Ca}^{0}$, (**b**) ${V}_{Zr}^{0}\,$and (**c**) ${V}_{O}^{0}\,$vacancies. The unrelaxed atomic positions are represented by solid spheres, while the relaxed atomic positions after the minimization of forces are masked onto these spheres using black rings. A black ring completely encompassing a solid sphere (as an outline) means that the atomic position does not change upon relaxation. Ca, Zr and O atoms are represented by green, gray and red spheres, respectively, while the vacancy site is depicted by a yellow sphere.

Upon introducing non-stoichiometry in CZO, in form of vacancy defects, the bulk coordinations of O_1_ and O_2_ atoms with Ca and Zr atoms are partially eliminated. In order to obtain a stable atomic configuration, it is necessary to minimize the non-zero forces acting on atoms neighboring the vacancy site. Figure 3 displays the positions of atoms around the vacant Ca, Zr and O sites before (coloured spheres) and after (black circles) the relaxation of the internal geometry of defective supercells of CZO. The off-centering of a black ring from a coloured sphere demonstrates the movement of atoms from their ideal atomic positions of pristine CZO when the structure is fully relaxed. Figure 3(a) clearly shows that in the case of a calcium or zirconium vacancy the neighboring Zr and Ca atoms, respectively, are attracted towards the vacancy site due to the eliminated repulsive electrostatic forces between the two cation sites. Contrary to that, the oxygen atoms exhibit outward relaxation when a Ca or Zr site is vacated. It is also evident that the vacant oxygen site in Fig. 3(c) results in lowest movement of the neighboring atoms.

**Figure 4:**
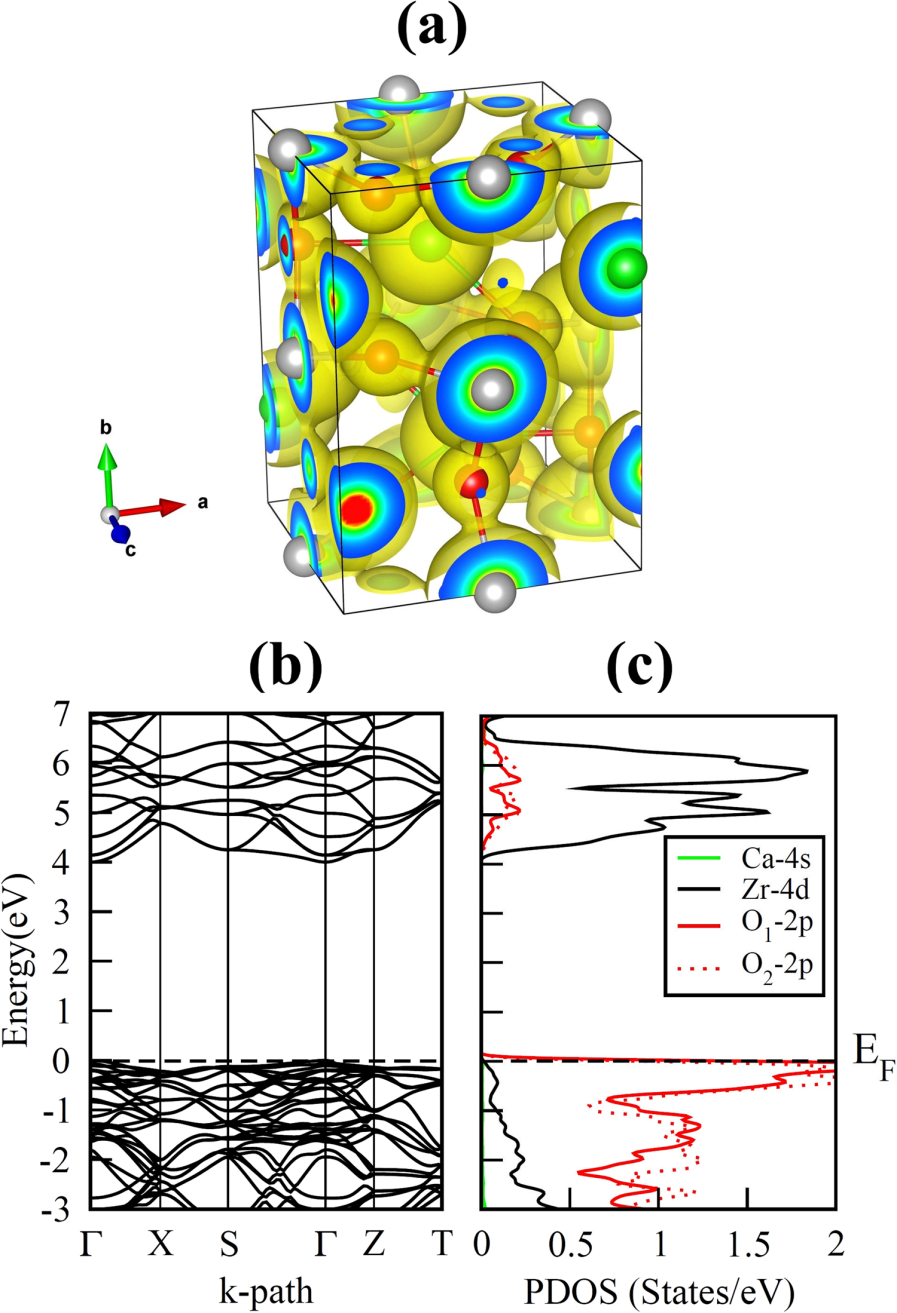
The calculated (**a**) electron-density distribution, (**b**) electronic band structure and (**c**) partial density of states of GGA optimized pristine orthorhombic unit cell of CaZrO_3_. For the electronic-density distribution an isosurface level of 0.3 $a.u{.}^{-3}\,$is used.

In order to understand the above-mentioned changes in atomic positions resulting from vacant cation and oxygen site, it is useful to compare the bonding properties of Ca/Zr atoms with the O atoms in pristine and intrinsic vacancy defect containing CZO. For this reason we have computed the 3-dimensional (3D) electron-density distribution in pristine orthorhombic unit cell of CZO which is displayed in Fig. 4(a). In addition, the effective Bader charges (*e*)^51^ are also calculated for a quantitative analysis of boning properties. Table 3 presents the effective Bader charges for the Ca, Zr and O atoms of pristine CZO and the atoms neighboring a vacancy site in defective supercell. It is clear from Fig. 4(a) that the bond between Zr and O atoms has a strong covalent nature, while ionic character of bonding prevails in the Ca–O bond. The effective Bader charges listed in Table 3 support these findings since the *e* value for Ca in pristine CZO is closer to its ionic limit (22% less than 2*e*) as compared to Zr (36% less than 4*e*). The covalent nature of Zr–O bond indicates that ${V}_{O}^{0}\,$would lead to larger delocalization of Zr charge density. Conversely, the incorporation of Zr vacancy would yield large charge localization for O_1_ and O_2_. The calculated changes in effective Bader charges of Zr and O_1_/O_2_ for oxygen and zirconium vacancy, respectively, support the above-mentioned charge redistribution. The comparison of the Zr Bader charges near the vacant apical and equatorial oxygen atoms reveals that an O_2_ vacancy results in slightly more charge delocalization. These findings are in accordance with previous theoretical investigations^52^.

**Table 3:** Estimated effective Bader charges, *e*, of Ca, Zr and O atoms in pristine CaZrO_3_ and supercells containing isolated ${V}_{X}^{0}$.

Supercell	Ca	Zr	O_1_	O_2_
Pristine CZO	1.549	2.554	−1.384	−1.365
Ca vacancy	—	2.572	−1.240	−1.229
Zr vacancy	1.545	—	−1.142	−1.063
O_1_ vacancy	1.528	1.722	—	−1.358
O_2_ vacancy	1.486	1.425	−1.446	—

**Figure 5:**
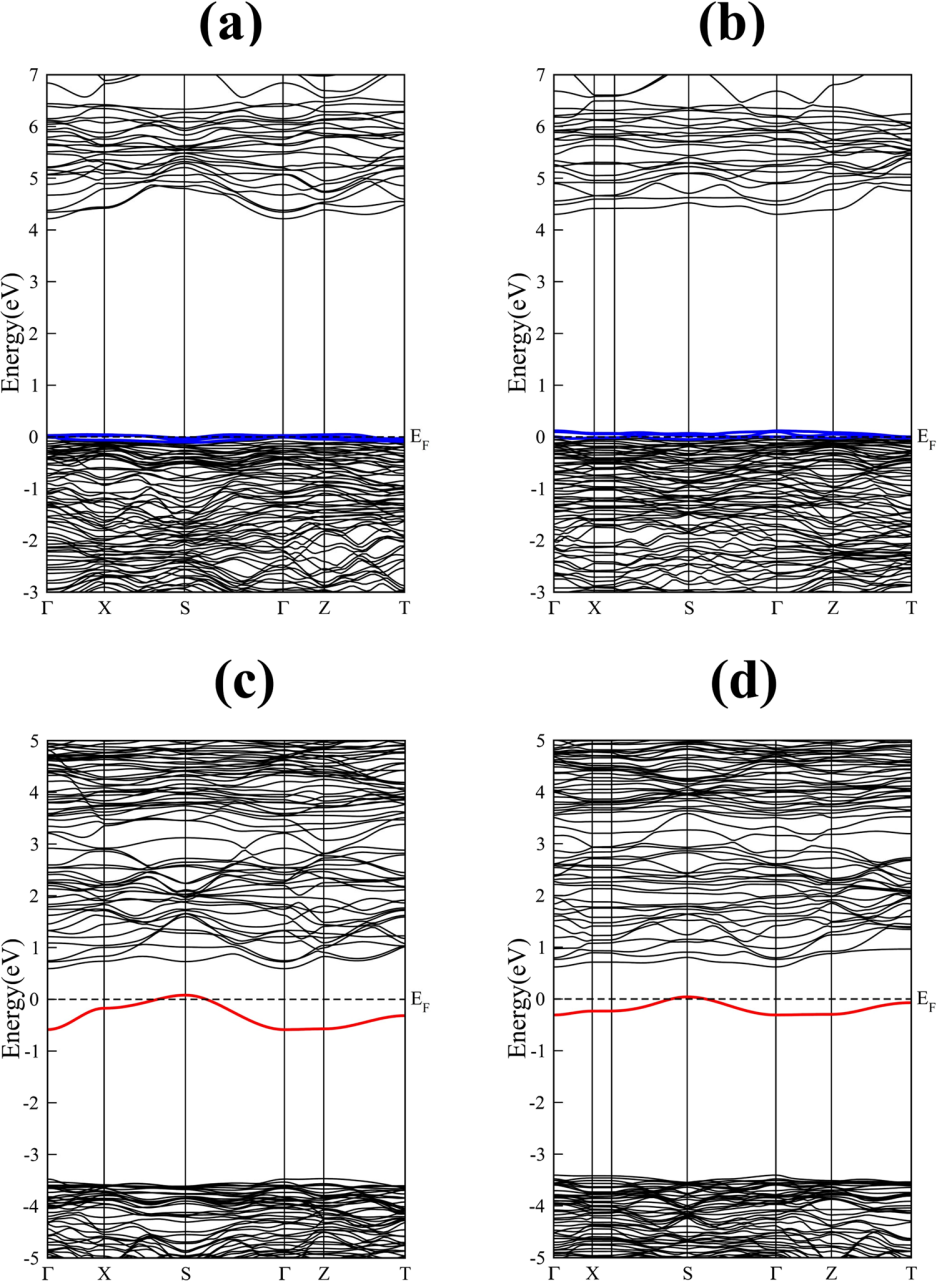
The calculated electronic band structures of charge-neutral (**a**) Ca, (**b**) Zr, (**c**) O_1_ and (**d**) O_2_ vacancy containing 2 × 1 × 2 supercells of CaZrO_3_. The Fermi level, occupied defect levels and unoccupied defect levels are indicated by dashed black line, solid red line and solid blue lines, respectively.

From the calculated electronic band structure of pristine CZO (Fig. 4(b)), we find that both the CBM and valence band maximum (VBM) are located at the Γ symmetry point; such that the occupied O-*2p* orbitals at the upper edge of the valence band and unoccupied Zr-*4d* orbitals at the lowest edge of the conduction band are separated by a direct fundamental energy band gap *E*_*g*_ = 4.003 eV. The calculated band gap is close to a previous GGA study^27^, however it is underestimated when compared with experimental value (1.497 eV less than the band gap of CZO reported in ref. 53). In spite of the underestimation of the band gap by the GGA functional, these results do not restrict us from a qualitatively exploration of the electronic properties of non-stoichiometric CZO^54^. Analysis of the partial density of states (PDOS) plots shown in Fig. 4(c) reveal that the upper valence band is predominantly made up of occupied anion *2p* orbitals with only minor differences between the contribution of O_1_-*2p* and O_2_-*2p* orbitals. The valence band of CZO also shows hybridization of the O-*2p* and Zr-*4d* orbitals which increases on going to lower (more negative) energies. On the other hand, the conduction band of CZO is dominated by the unoccupied Zr-*4d* orbitals. In case of defective supercells, the incorporation of ${V}_{Ca}^{0}\,$or ${V}_{Zr}^{0}\,$in CZO results in acceptor-like defect levels just above the bulk VBM, having an unoccupied O-*2p* character (Fig. 5(a and b). It is clear from Fig. 5(a and b) that a maximum localization of charge is achieved at occupied O site near the ${V}_{Zr}^{0}$. This suggests that presence of electrons in acceptor-like defect levels (e.g. a ${V}_{Zr}^{4-}\,$vacancy) would raise these levels closer to the CBM of bulk CZO as compared to charged Ca vacancies. This finding is in accord with the data listed in Table 3 where *e* of oxygen atom near ${V}_{Zr}^{0}\,$shows large reduction.

The band structures of Ca and Zr deficient supercell presented in Fig. 5(a) and (b), respectively, show that triply degenerate anion-orbital like states (represented by blue colour) cross the *E*_*F*_. These states are positioned slightly higher on the energy scale for the case of ${V}_{Zr}^{0}\,$ indicating that charge neutral Zr vacancy is a relatively deep acceptor as compared to charge neutral Ca vacancy. On the other hand, ${V}_{O}^{0}\,$give rise to an occupied donor level close to the unoccupied CBM of bulk CZO. At the Γ symmetry point, the occupied defect level resulting from the incorporation of O_1_/O_2_ vacancy (represented by red colour in Fig. 5(c,d)) is located 2.889 eV/3.095 eV above the bulk-like O-*2p* orbitals. On pushing the calculated CBM 1.497 eV (i.e. the difference of PBE and experimental band gap) higher along the energy scale, the ${V}_{O}^{0}\,$defect level would be positioned 4.386 eV/4.592 eV above the bulk VBM and 1.114 eV/0.908 eV below the CBM.

### Formation Energetics

The formation energies of ${V}_{Ca}^{q}$, ${V}_{Zr}^{q}\,$and ${V}_{O}^{q}\,$ vacancies in CZO have been estimated using the formula^46^

7 $$\Omega [n{X}^{q}]=E[n{X}^{q}]-{E}^{CaZr{O}_{3}}-n{\mu }_{x}+q({E}_{F}+{E}_{VBM})$$

The minimum total energies of bulk CZO and ${V}_{X}^{q}\,$containing supercells are represented by ${E}^{CaZr{O}_{3}}\,$and $E[n{X}^{q}],\,$respectively, *n* is the number of *X* atoms removed from a supercell and *μ*_*x*_ is the chemical potential of the species *X*. For comparing the stability of different types of vacancy defects in CZO, we use the chemical potential coordinates defined in Fig. 2. The term *E*_*F*_ + *E*_*VBM*_ in Equation 7 references the Fermi level with respect to energy of the VBM in defective supercell^46^. Since finite size of the supercell under periodic boundary conditions makes the *E*_*VBM*_ in defective supercell is different from *E*_*VBM*_ of the pristine CZO, we have computed the average electrostatic potentials, V_avg._, at the occupied sites of vacancy type atom in pristine (*pr*.) and vacancy containing (*de*.) supercell (far away from the vacancy site) and the difference of these electrostatic potentials (ΔV_avg._ = V^*pr*.^_avg._−V^*de.*^_avg._) has been added into *E*_*VBM*_. Moreover, the formation energies of oxygen vacancies have been corrected for the underestimation of band gap resulting from the use of GGA functional. This has been accounted for by using the band gap correction^54^ where difference of experimental and GGA band gap (*ΔE*_*g*_ = 1.497 eV) times *m* (number of electrons) has been added into the calculated $\Omega [n{O}^{q}]\,$values computed using Equation 7. This simple bad gap correction in the formation energies calculated using semilocal functionals has been found to provide reasonable agreement between theoretically computed defect levels for isolated oxygen vacancies and experimental observations^46, 54^. It is worth pointing out here that hybrid DFT functionals can improve the evaluations of electronic properties in wide band gap complex oxides. However, the extremely high computational costs of hybrid DFT functionals^55^ and the need to still adopt band gap correction for a relatively smaller underestimation of the band gap^56^ allows us to use PBE-GGA for evaluating formation energetics of CZO using the all-electron FP-LAPW method.

**Figure 6:**
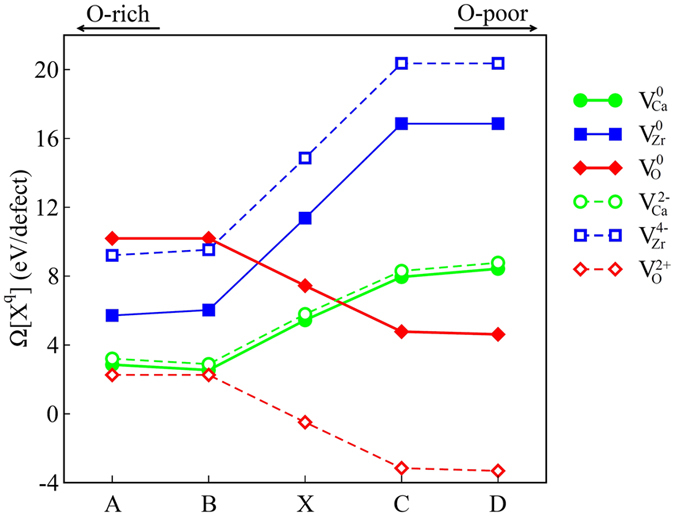
The calculated formation energies (eV/defect) of ${V}_{Ca}^{q}$, ${V}_{Zr}^{q}\,$and ${V}_{O}^{q}\,$ vacancies in CaZrO_3_ at stability coordinates defined in Fig. 2.

Finally, the image charge corrections for charged vacancies proposed by Lany and Zunger^57^ have been computed from

8 $${\rm{\Delta }}{E}_{ICC}=\frac{2}{3}\frac{{q}^{2}{\alpha }_{M}}{2\varepsilon L}$$

which are added to the calculated formation energies. In Equation 8, *α*_*M*_ is the Madelung constant of a perovskite-like structure^58^, *ε* is the experimental dielectric constant of CZO^59^ and *L* is edge of the structure used for simulating charged vacancy defects in CZO. The calculated formation energies at points A, B, C, D and X for neutral and fully-charged vacancy defects are displayed in Fig. 6. The formation energies of intermediate charge states of the vacancy defects are also computed, however, these values are not shown in Fig. 6. It is important to point out here that our calculations reveal that the defect formation energy of charge neutral O_2_ vacancy and O_1_ vacancy differ only by 0.015 eV. Since the band gap correction (*mΔE*_*g*_) and the image charge correction of Equation 8 are constant quantities, in this section we only discuss the relative stability of oxygen vacancies in terms of the values obtained for O_1_ vacancies.

For the case of neutral vacancy defects, our results reveal that ${V}_{Ca}^{0}\,$has the lowest formation energy ($\Omega [C{a}^{0}]$ = 2.857 eV) at point A (O-rich condition) followed by ${V}_{Zr}^{0}$($\Omega [Z{r}^{0}]$ = 5.715 eV) and ${V}_{O}^{0}$($\Omega [{O}^{0}]$ = 10.191 eV). The trend remains unchanged for point B (${\rm{\Omega }}[C{a}^{0}]$ = 2.540 eV and ${\rm{\Omega }}[Z{r}^{0}]$ = 6.033 eV) where ${V}_{Ca}^{0}\,$ is still more stable than other neutral vacancies. Even though ${V}_{O}^{0}\,$ becomes more stable than ${V}_{Zr}^{0}\,$ at point X,${V}_{Ca}^{0}\,$ still remains the most favorable form of neutral vacancy defect in CZO at points X which is in excellent agreement wh experimentally observed mixed *p*-type and ionic conductivity in acceptor-doped CZO that depends on Ca content^23^. At point C/point D, ${V}_{O}^{0}\,$ is found to be more stable than ${V}_{Zr}^{0}\,$ and ${V}_{Ca}^{0}$ with ${\rm{\Omega }}[{O}^{0}]$ = 4.77 eV/4.618 eV. From Fig. 6, it is evident that the value of ${\rm{\Omega }}[{O}^{2+}]\,$ is less than the formation energies of all other vacancy defects for point A through point D, showing that the fully charged oxygen vacancy can be easily incorporated in CZO. The formation energies of fully ionized Ca and Zr vacancies, on the other hand, are found to be larger than their charge neutral counterparts. These larger formation energies of negatively charged cation defects can be attributed to their electron scavenger nature as these vacancies tend to lower the energy of the supercell. Contrary to that, the creatn of ionized oxygen vacancies adds additional electrons to the system, compensating the occupied donor defect levels shown in Fig. 5(c) and (d), and increasing the energy. Since incorporation of charge neutral oxygen vacancies in CZO results in the appearance of an occupied donor level below the CBM, the singly ionized oxygen vacancy ${V}_{O}^{1+}\,$ would result in further deepening of this donor-like level located at 1.520 eV below the CBM and occupied by one electron. On the other hand, ${V}_{O}^{2+}\,$ will result in an empty defect level located 1.548 eV below the CBM. Comparison of the calculated formation energies and the trends for oxygen vacancy concentrations, [${V}_{O}^{q}$], in ceramics^60^ allow us to predict that under O-poor conditions the equilibrium concentration of oxygen vacancies would satisfy [${V}_{O}^{1+}$] ≫ [${V}_{O}^{2+}$], [${V}_{O}^{0}$] for low temperatures, while [${V}_{O}^{2+}$] ≫ [${V}_{O}^{1+}$], [${V}_{O}^{0}$] will be satisfied for high temperatures regime.

**Figure 7:**
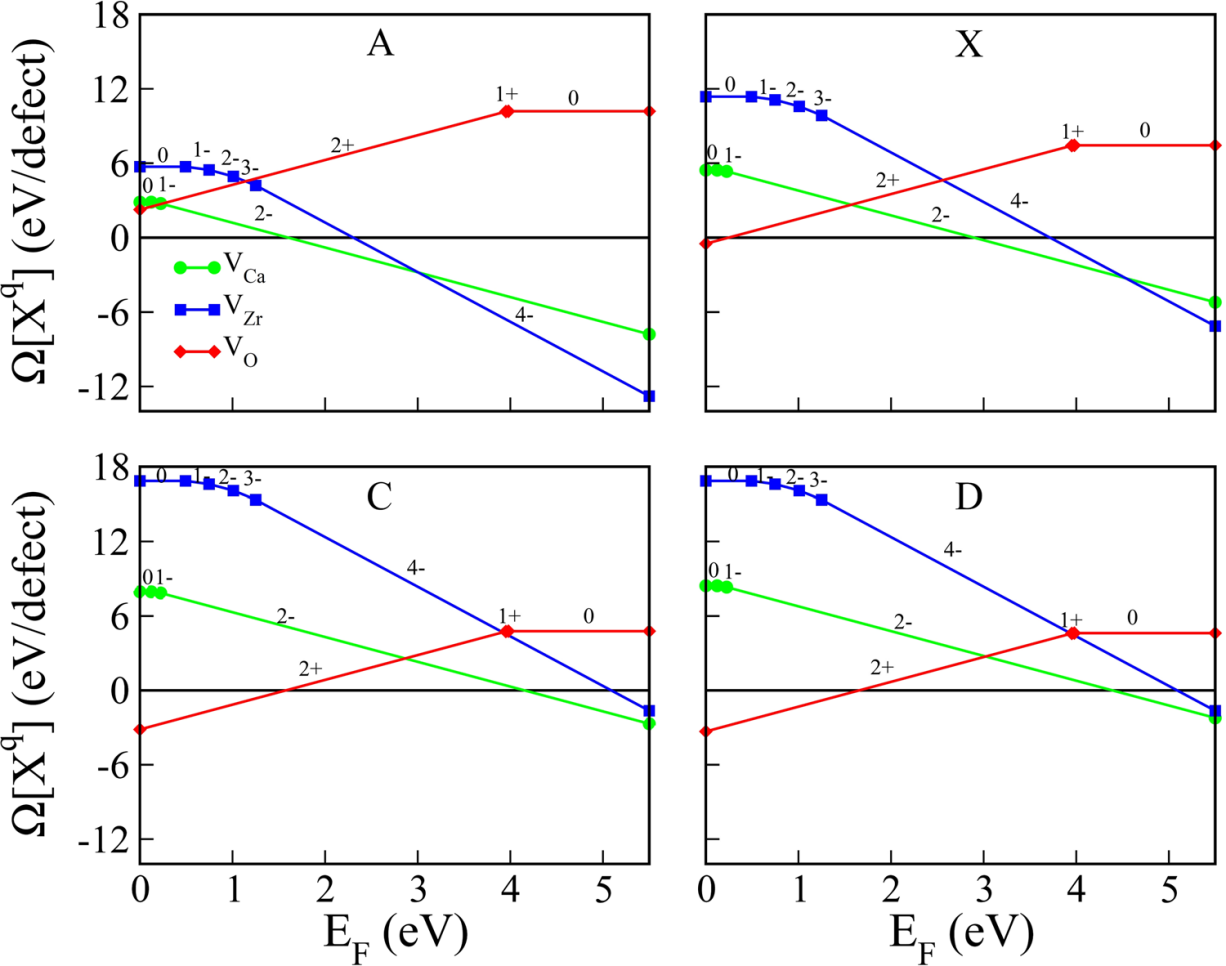
The variation of the ${\rm{\Omega }}[{X}^{q}]\,$with *E*_*F*_ for selected stability points. Signed numbers over the lines indicate the charge states of the vacancy defect.

From the calculated ${\rm{\Omega }}[C{a}^{2-}]$, ${\rm{\Omega }}[{O}^{4-}]\,$ and ${\rm{\Omega }}[{O}^{2+}]\,$ we have obtained the full and partial Schottky reaction energies (ζ)^61^. The average Ca-partial and Zr-partial Schottky ζ are found to be 2.632 eV and 3.643 eV, respectively, where larger values of Zr-partial Schottky ζ supports easy incorporation of Ca vacancy in CZO^26^. On the other hand, the full Schottky ζ is found to be 3.839 eV. Figure 7 shows the variation of ${\rm{\Omega }}[{X}^{q}]\,$with *E*_*F*_ = 0 eV to *E*_*F*_ = 5.5 eV. In all cases ${V}_{O}^{2+}\,$has the smallest formation energy at *E*_*F*_ = 0 eV, while ${V}_{Zr}^{4-}\,$has the smallest value of $\Omega [{X}^{q}]\,$at *E*_*F*_ = 5.5 eV at points A and point X. At certain values of *E*_*F*_ the crossover of the horizontal axis by ${\rm{\Omega }}[{X}^{q}]\,$gives the pinning energies (*E*_*pin*_)^62^ which ensures that the *E*_*F*_ in CZO is not positioned too close to the CBM and VBM. The electronic band structures and the variation in calculated ${\rm{\Omega }}[{X}^{q}]\,$for intrinsic vacancy defect in CZO (Figs 5 and 6), reveal that under the metal-poor (oxidation) conditions charge neutral Ca vacancies could give rise to *p*-type conductivity in CZO. However, the small value of Ca-partial Schottky ζ suggests that self-compensation between fully charged Ca and O vacancies would work to decrease the *p*-type electrical conductivity in Ca deficient CZO. Although a concrete conclusion regarding the introduction of hole-doped state in CZO can not be established without comparison with other intrinsic (e.g. interstitial and anti-site defects) and extrinsic (cation dopants) point defects, the case of fully charged ${V}_{O}^{2+}\,$at point A shows no Fermi level pinning indicating that both un-doped and acceptor-doped CZO can accommodate isolated Ca vacancies and *p*-type conductivity under extreme reduction condition.

### Clustering of Oxygen Vacancies

The simplest and the most abundant form of point defects in perovskite oxides are the isolated intrinsic vacancies which are necessitated by the thermodynamical requirement of increasing the entropy of a chemical system^63^. In the past, various studies have shown that unusual electronic and transport properties of transition metal perovskite oxides can not be attributed to isolated intrinsic vacancy defects^64^. In these situations, other forms of native point defect (such as self-interstitial or anti-site defects) are the possible suspects, however, large distortion in the crystal geometry and the resulting decrease in chemical stability^63^ are generally not responsible for enhancing/degrading electronic and transport properties of bulk CZO. To this end, clustering of oxygen vacancies in perovskite oxides has been found responsible for greatly influencing the electronic properties. In fact, transmission electron microscopy and complementary independence spectroscopy studies have confirmed the role of oxygen vacancy clustering in the unusual properties of transition metal perovskite oxides^65, 66^. Since our results clearly show that ${V}_{O}^{0}\,$is the most favorable form of charge neutral intrinsic vacancy defects in CZO at both point C and D, in this section we explore the possibility of realizing oxygen vacancy clustering in CZO under the extreme reduction condition.

**Figure 8:**
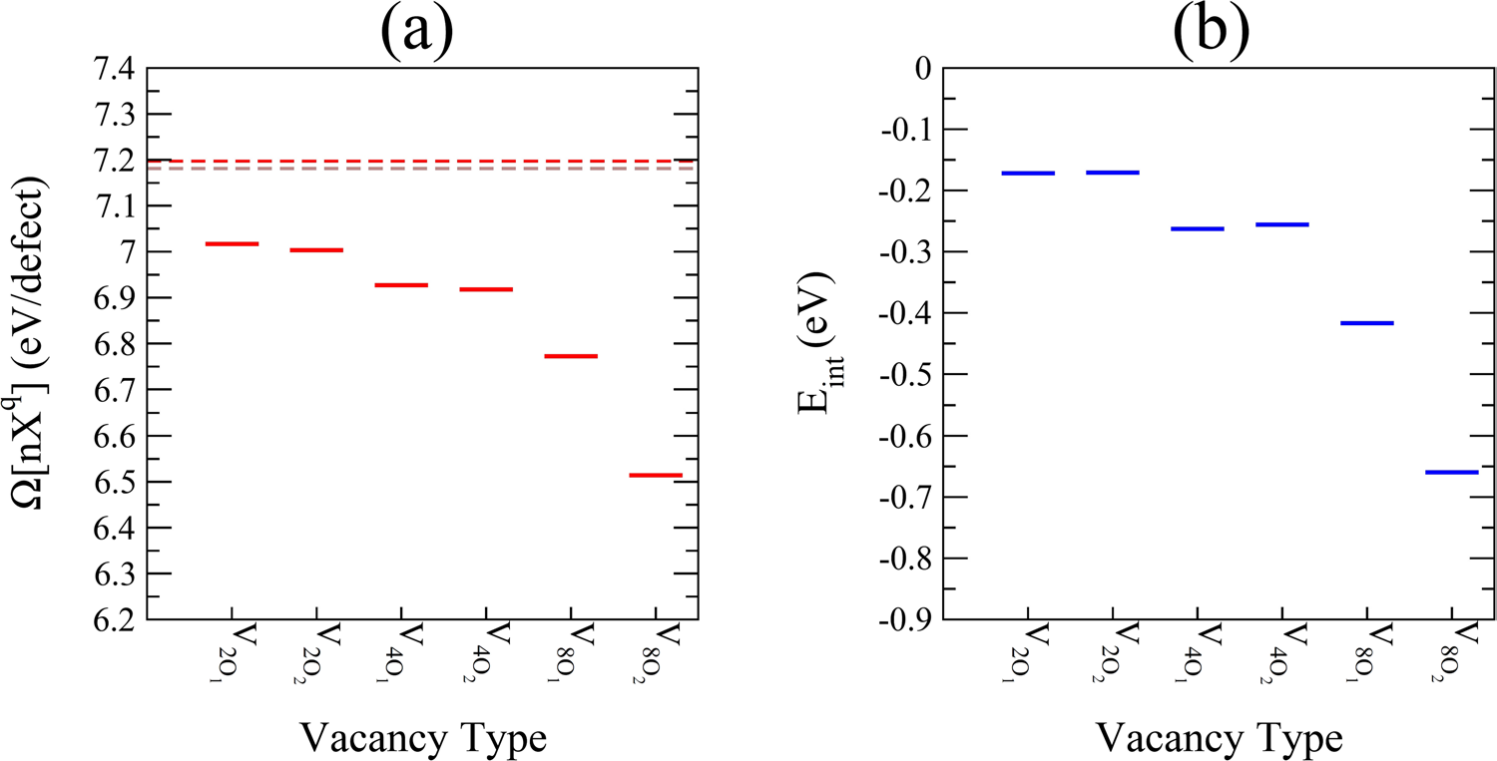
The calculated (**a**) ${\rm{\Omega }}[n{X}^{q}]$(eV/defect) at point D and (**b**) *E*_*int*_ (eV) for different cases of ${V}_{O}^{0}\,$clustering cases explained in the text. For the sake of comparison, the formation energies of O_1_ (dashed red line) and O_2_ (dashed brown line) vacancies are also presented in (**a**).

We have calculated ${\rm{\Omega }}[n{X}^{q}]\,$for oxygen vacancy clustering (as explained in Method of Calculation section) using Equation 7 at the extreme reduction condition (Fig. 8(a)) along with the formation energies of isolated neutral O_1_ (red dashed line) and O_2_ (brown dashed line) vacancies. It is evident that the charge-neutral *V*_8*O*2_ has the lowest formation energy values which is 0.638 eV less than ${\rm{\Omega }}[{{O}_{2}}^{0}]$. A close inspection of Fig. 8(a) reveals that the removal of 2 and 4 oxygen atoms from Ca_8_O_8_ and Zr_8_O_16_ layers (i.e.${V}_{2{O}_{1/2}}\,$and ${V}_{4{O}_{1/2}}$) of the 2 × 1 × 2 supercell results in similar difference in the formation energy values of O_1_ and O_2_ vacancies (*V*_*O*2_ having lower formation energy value) as was the case of isolated neutral O_1_ and O_2_ vacancies. This trend, however, changes for the case of ${V}_{8{O}_{1/2}}\,$where a larger (0.258 eV) reduction in the formation energies of clustered O_1_ and O_2_ vacancies is achieved. This significant decrease in $\Omega [n{X}^{q}]\,$of ${V}_{8{O}_{1/2}}\,$hints at the attractive interaction in O vacancy clustered in a 2 × 1 × 2 supercell of CZO. This attractive interaction can be quantitatively determined from the interaction energy which is given by

9 $${E}_{{\rm{int}}}=(E[n{O}^{0}]-{E}^{CaZr{O}_{3}})-n(E[{O}^{0}]-{E}^{CaZr{O}_{3}})$$

Figure 8(b) displays the calculated *E*_*int*_ for all cases of oxygen vacancy clustering where one can see that higher concentration *V*_*O*1_ and *V*_*O*2_ in the Zr_8_O_16_ layer of CZO are relatively more favorable. Referring back to the effective Bader charges in case of isolated metal atom vacancies listed in Table 3 and the strong hybridization of O-*2p* and Zr-*4d* orbitals just below *E*_*F*_ shown in Fig. 4(c), it is clear that increasing oxygen vacancies clustering in the Zr_8_O_16_ layer would lead to increase in charge delocalization which causes more attractive interaction among the Zr atoms in the layer. Moreover, a comparison of the defect formation energies presented in Figs 6 and 8(a) allows us to speculate that clustering of charged oxygen vacancies in the Zr_8_O_16_ layer of CZO can be realized. The above findings are encouraging in view of using acceptor-doped CZO for high protonic conduction where proton trapping^67^ causing major hindrance in long-range conduction can be effectively reduced by the formation of charged oxygen vacancy–acceptor clusters^62^.

**Figure 9:**
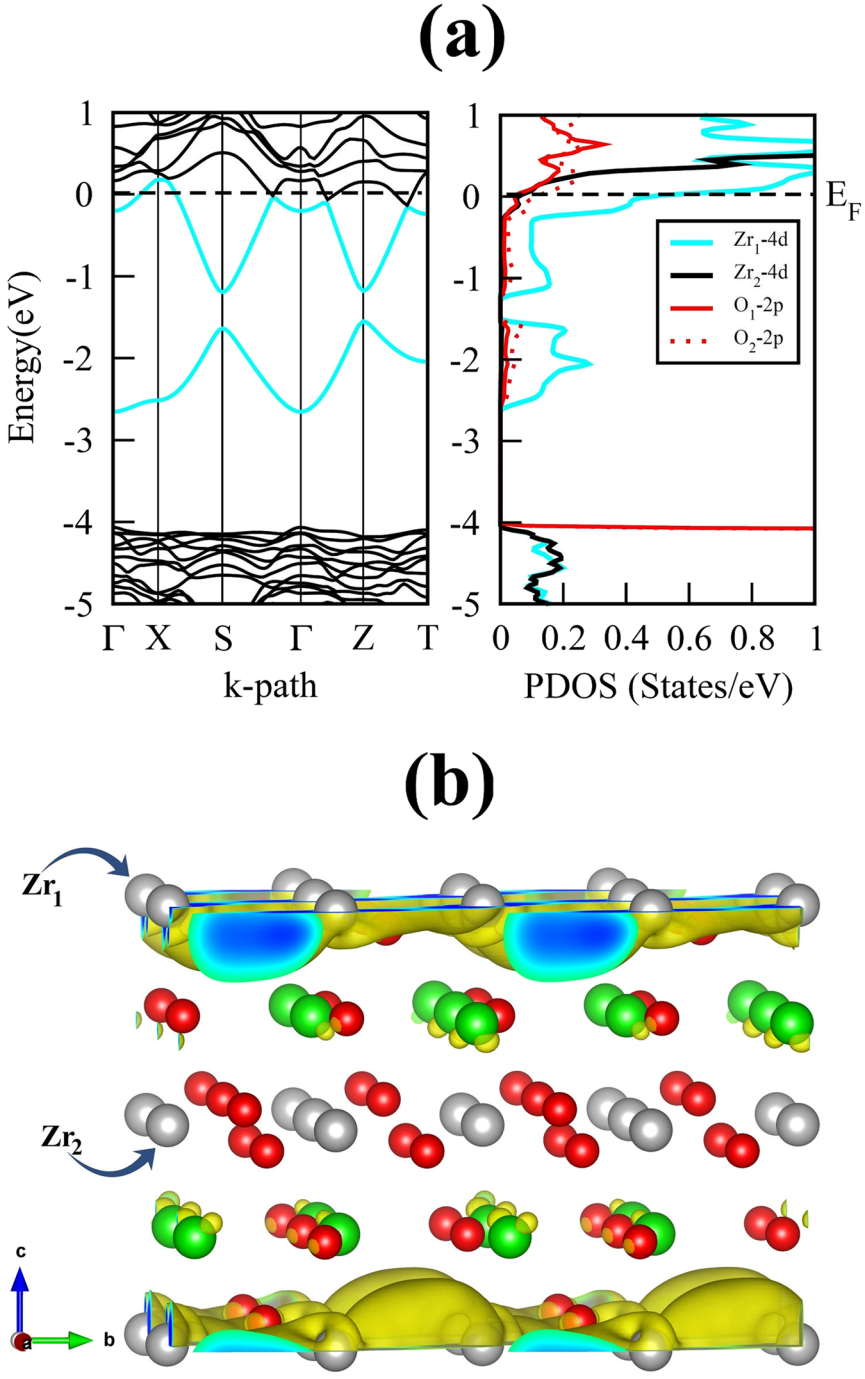
The calculated electronic properties and 3D valence charge density isosurfaces for ${V}_{8{O}_{2}}\,$containing 2 × 1 × 2 supercell of CaZrO_3_. The 3D valence charge density isosurfaces are calculated with an isosurface level of 0.005 *a.u*.^*−3*^ for the electronic states ranging from −1.30 eV up to the *E*_*F*_.

For exploring the electronic properties of oxygen vacancy clustering in CZO, we have computed the electronic DOS for all the oxygen vacancy clattering cases. The calculated electronic band structure and DOS for ${V}_{2{O}_{1}}$, ${V}_{2{O}_{2}}$, ${V}_{4{O}_{1}}$, ${V}_{4{O}_{2}}\,$ and ${V}_{8{O}_{1}}$ (not shown here) reveal deep donor-like levels of these charge neutral oxygen vacancy clustering cases similar to the ones shown in Fig. 5(c) and (d). In contrast, this donor-like level shifts into the CBM for the case of ${V}_{8{O}_{2}}$(Fig. 9(a)). The shifting of this defect level into the conduction band results from large charge delocalization from the zirconium atoms residing in defective Zr_8_O_16_ layer (Zr_1_: residing in the Zr_8_O_16_ layer containing the ${V}_{8{O}_{2}}\,$vacancy) as shown in Fig. 9(a). The confinement of the delocalized charge in ${V}_{8{O}_{2}}\,$containing Zr_8_O_16_ layer of CZO (Fig. 9(b)), therefore, allows the Zr_1_-*4d* states to move across *E*_*F*_. By using a 2 × 2 × 2 supercell we found that the presence of occupied states in CBM persists for charge neutral ${V}_{8{O}_{2}}\,$clustering in CZO and is independent of the increasing number of defect free Ca_8_O_8_ and Zr_8_O_16_ layers between two ${V}_{8{O}_{2}}\,$containing Zr_8_O_16_ layers.

Figure 9(b) provide us a clear picture of the origin of this possible “*n*-type” character where the 3D charge density isosurfaces show that the valence charge density of Zr_1_ atom of the defective Zr_8_O_8_ layer is highly delocalized as compared to the valence charge density of Zr_2_ atom of defect free Zr_8_O_16_ layer. To further understand the changes in charge distribution we have computed the effective Bader charges for the case of ${V}_{8{O}_{2}}\,$containing CZO. The effective Bader charges of Ca atom residing in the defect free Ca_8_O_8_ layer, Zr_2_ atom residing in the defect free Zr_8_O_16_ layer and Zr_1_ atom residing in the ${V}_{8{O}_{2}}\,$containing Zr_8_O_8_ layer of CZO become 1.461 *e*, 2.544 *e* and 1.411 *e*, respectively. On the other hand, the effective Bader charge of Zr atoms residing in the defect free Zr_8_O_16_ layer, Ca_2_ atom residing in the defect free Ca_8_O_8_ layer and Ca_1_ atom residing in the ${V}_{8{O}_{2}}\,$containing Ca_8_O_0_ layer change to 2.460 *e*, 1.584 *e* and 1.324 *e*, respectively. This confirms the large charge delocalization in case of ${V}_{8{O}_{2}}\,$containing CZO which causes Zr_1_-*4d* states to move across *E*_*F*_. Although we have only considered neutral oxygen vacancy clustering in the present work, an easier incorporation of charged oxygen vacancy clustering in CZO can also be deduced from the formation energies presented in Fig. 6. These findings support the use of acceptor-doped CZO for high protonic conduction where proton trapping can be avoided by intentionally clustering charged oxygen vacancies (e.g. ${V}_{O}^{1+}$) in the Zr_8_O_16_ layer of CZO^68^. The clustering of charged oxygen vacancies would certainly eliminate the charge delocalization and *n*-type nature of CZO shown in Fig. 9, however, this reduction clearly supports the decreased electronic conductivity and increased ionic conduction in Y_2_O_3_ (etc.)-doped CZO which makes calcium zirconate a potential solid-state electrolyte materials for solid oxide fuel cell technology^23, 24^.

## Conclusions

In summary, we have employed first-principles calculations for investigating the influence of isolated ${V}_{Ca}^{q}$, ${V}_{Zr}^{q}\,$ and ${V}_{O}^{q}\,$ vacancies and oxygen vacancy clustering on the electronic structure of orthorhombic CZO. Our results reveal that pristine as well as low concentration of charge neutral oxygen vacancy containing CZO are insulating. On the other hand, charge neutral Ca and Zr vacancy containing CZO are found to be hole-doped systems, where low formation energies of Ca vacancy confirms the contribution of ${V}_{Ca}^{0}\,$in experimentally observed mixed *p*-type and ionic conduction behaviour of CZO. For the case of neutral vacancy defects, calcium/oxygen vacancies are the most abundant form of vacancy defects in CZO under oxidation/reduction condition. We find that fully charged oxygen vacancies are the most favorable as compared to all other types and charge states of intrinsic vacancy defects in CZO. The calculated values of Schottky ζ permit us to predict that non-stoichiometric CZO could allow tunable *p*-type conductivity. It is shown that a high concentration of O vacancies can be experimentally realized in the Zr_8_O_16_ layer of CZO which highlights potential utilization of acceptor-doped CZO for high protonic conduction where proton trapping can be avoided by means of introducing charged oxygen vacancies around the dopant site. The wide range of possibilities in tailoring the electronic structure of CZO by means of intrinsic vacancy defects makes it attractive for electronic, electrical and optical devices.

## Electronic supplementary material

Dataset 1
