# Supplementary material for: Evaluation of thermodynamics, formation energetics and electronic properties of vacancy defects in CaZrO3
Source: Sci Rep. 2017 Aug 16;7:8439. doi: 10.1038/s41598-017-08189-2 (PMC5559480; doi:10.1038/s41598-017-08189-2)
Supplement: Supplementary file 1 — Dataset 1 [file 41598_2017_8189_MOESM1_ESM.doc]

**Evaluation of thermodynamics, formation energetics and electronic properties of vacancy defects in CaZrO_3_**

Syed Muhammad Alay-e-Abbas, Safdar Nazir, Stefaan Cottenier, Ali Shaukat

**Supplementary information on PBE-GGA optimized geometries of pristine and vacancy defect containing supercells (Crystallographic Information Files)**

**Pristine CaZrO_3_**

data_Pristine_CaZrO_3_

_cell_length_a 5.806908

_cell_length_b 8.072617

_cell_length_c 5.620258

_cell_angle_alpha 90.000000

_cell_angle_beta 90.000000

_cell_angle_gamma 90.000000

_cell_measurement_temperature 0.0

_diffrn_ambient_temperature 0.0

_symmetry_space_group_name_H-M 'Pnma '

_symmetry_space_group_number 62

_refine_date '11-12-2016'

_refine_method 'generated from Wien2k code'

_refine_special_details

;

Structure converted from Wien2k struct file, Version 9.1

File Name /home/alay-e-abbas/Desktop/Optimized_Structures_CZO/struct/CaZrO3/CaZrO3.struct

Title 'CZO_optimized'

;

loop_

_symmetry_equiv_pos_as_xyz

+x,+y,+z

-x+1/2,-y,+z+1/2

-x,+y+1/2,-z

+x+1/2,-y+1/2,-z+1/2

-x,-y,-z

+x+1/2,+y,-z+1/2

+x,-y+1/2,+z

-x+1/2,+y+1/2,+z+1/2

loop_

_atom_site_label

_atom_site_type_symbol

_atom_site_fract_x

_atom_site_fract_y

_atom_site_fract_z

_atom_site_U_iso_or_equiv

Ca001 Ca 0.54996900 0.25000000 0.48678900 0.05000000

Zr002 Zr 0.50000000 0.00000000 0.00000000 0.05000000

O0003 O 0.45931900 0.25000000 0.89350600 0.05000000

O0004 O 0.79922200 0.05660200 0.19837700 0.05000000

#End data_Pristine_CaZrO_3_

**Ca_15_Zr_16_O_48_**

data_Ca_15_Zr_16_O_48_

_cell_length_a 11.613816

_cell_length_b 11.240515

_cell_length_c 8.072617

_cell_angle_alpha 90.000000

_cell_angle_beta 90.000000

_cell_angle_gamma 90.000000

_cell_measurement_temperature 0.0

_diffrn_ambient_temperature 0.0

_symmetry_space_group_name_H-M 'Pm '

_symmetry_space_group_number 6

_refine_date '11-12-2016'

_refine_method 'generated from Wien2k code'

_refine_special_details

;

Structure converted from Wien2k struct file, Version 9.1

File Name /home/alay-e-abbas/Desktop/Optimized_Structures_CZO/struct/Ca15Zr16O48/Ca15Zr16O

Title 'CZO_optimized'

;

loop_

_symmetry_equiv_pos_as_xyz

+x,+y,+z

+x,+y,-z

loop_

_atom_site_label

_atom_site_type_symbol

_atom_site_fract_x

_atom_site_fract_y

_atom_site_fract_z

_atom_site_U_iso_or_equiv

Ca001 Ca 0.99782699 0.00165061 0.00000000 0.05000000

Ca002 Ca 0.49775859 0.99802461 0.00000000 0.05000000

Ca003 Ca 0.55008641 0.48341114 0.50000000 0.05000000

Ca004 Ca 0.54834473 0.99197262 0.50000000 0.05000000

Ca005 Ca 0.80291197 0.24738816 0.50000000 0.05000000

Ca006 Ca 0.25606474 0.23761000 0.00000000 0.05000000

Ca007 Ca 0.05059078 0.48608953 0.50000000 0.05000000

Ca008 Ca 0.04598880 0.98834598 0.50000000 0.05000000

Ca009 Ca 0.30525536 0.25004105 0.50000000 0.05000000

Ca010 Ca 0.74970103 0.73262100 0.00000000 0.05000000

Ca011 Ca 0.80179000 0.75179868 0.50000000 0.05000000

Ca012 Ca 0.00115788 0.50146981 0.00000000 0.05000000

Ca013 Ca 0.49791416 0.50177794 0.00000000 0.05000000

Ca014 Ca 0.25047218 0.73658412 0.00000000 0.05000000

Ca015 Ca 0.30041637 0.75215706 0.50000000 0.05000000

Zr016 Zr 0.77045865 0.99471226 0.75381135 0.05000000

Zr017 Zr 0.02699471 0.24462105 0.75327111 0.05000000

Zr018 Zr 0.27494161 0.99453594 0.74970994 0.05000000

Zr019 Zr 0.53145628 0.24324672 0.75550081 0.05000000

Zr020 Zr 0.77352077 0.49114343 0.74827550 0.05000000

Zr021 Zr 0.02577350 0.74392890 0.75000516 0.05000000

Zr022 Zr 0.27644414 0.49356086 0.74821819 0.05000000

Zr023 Zr 0.52585842 0.74315531 0.74790391 0.05000000

O0024 O 0.80074084 0.46220765 0.00000000 0.05000000

O0025 O 0.01088550 0.19346566 0.50000000 0.05000000

O0026 O 0.75371359 0.04562873 0.50000000 0.05000000

O0027 O 0.53046475 0.29845248 0.00000000 0.05000000

O0028 O 0.29354349 0.44221723 0.00000000 0.05000000

O0029 O 0.50419968 0.19469703 0.50000000 0.05000000

O0030 O 0.25306515 0.04720537 0.50000000 0.05000000

O0031 O 0.00632582 0.68938478 0.50000000 0.05000000

O0032 O 0.50297240 0.68757690 0.50000000 0.05000000

O0033 O 0.25409334 0.54919974 0.50000000 0.05000000

O0034 O 0.04979550 0.29973141 0.00000000 0.05000000

O0035 O 0.79387085 0.93819835 0.00000000 0.05000000

O0036 O 0.75623337 0.54996374 0.50000000 0.05000000

O0037 O 0.54528230 0.79565465 0.00000000 0.05000000

O0038 O 0.29646460 0.93888452 0.00000000 0.05000000

O0039 O 0.04577820 0.79771917 0.00000000 0.05000000

O0040 O 0.61435213 0.08128606 0.79141974 0.05000000

O0041 O 0.67176828 0.34584466 0.68217180 0.05000000

O0042 O 0.92416920 0.39306300 0.30698368 0.05000000

O0043 O 0.88549560 0.13347578 0.21051086 0.05000000

O0044 O 0.12742815 0.09378684 0.81102588 0.05000000

O0045 O 0.17551079 0.34304168 0.69037728 0.05000000

O0046 O 0.42370200 0.39331923 0.30599628 0.05000000

O0047 O 0.37549593 0.14387020 0.19631228 0.05000000

O0048 O 0.62380109 0.59081511 0.80566656 0.05000000

O0049 O 0.67586738 0.83923285 0.69300596 0.05000000

O0050 O 0.92208170 0.89130925 0.30767308 0.05000000

O0051 O 0.87494746 0.64608237 0.19799872 0.05000000

O0052 O 0.12498378 0.59244837 0.80673640 0.05000000

O0053 O 0.17328678 0.84367718 0.69153980 0.05000000

O0054 O 0.42271852 0.89236400 0.30891353 0.05000000

O0055 O 0.37498141 0.64448842 0.19200960 0.05000000

#End data_Ca_15_Zr_16_O_48_

**Ca_16_Zr_15_O_48_**

data_Ca_16_Zr_15_O_48_

_cell_length_a 8.072617

_cell_length_b 11.240515

_cell_length_c 11.613816

_cell_angle_alpha 90.000000

_cell_angle_beta 90.000000

_cell_angle_gamma 90.000000

_cell_measurement_temperature 0.0

_diffrn_ambient_temperature 0.0

_symmetry_space_group_name_H-M 'P-1 '

_symmetry_space_group_number 2

_refine_date '11-12-2016'

_refine_method 'generated from Wien2k code'

_refine_special_details

;

Structure converted from Wien2k struct file, Version 9.1

File Name /home/alay-e-abbas/Desktop/Optimized_Structures_CZO/struct/Ca16Zr15O48/Ca16Zr15O

Title 'CZO_optimized'

;

loop_

_symmetry_equiv_pos_as_xyz

+x,+y,+z

-x,-y,-z

loop_

_atom_site_label

_atom_site_type_symbol

_atom_site_fract_x

_atom_site_fract_y

_atom_site_fract_z

_atom_site_U_iso_or_equiv

Ca001 Ca 0.30062988 0.99616431 0.30085578 0.05000000

Ca002 Ca 0.72797736 0.22902521 0.47004687 0.05000000

Ca003 Ca 0.74640535 0.00172917 0.22978957 0.05000000

Ca004 Ca 0.24195874 0.75730617 0.02389250 0.05000000

Ca005 Ca 0.24306753 0.49312709 0.27808633 0.05000000

Ca006 Ca 0.73611490 0.74468843 0.47366717 0.05000000

Ca007 Ca 0.76023713 0.50798081 0.22472398 0.05000000

Ca008 Ca 0.24201954 0.25332507 0.02569391 0.05000000

Zr009 Zr 0.00926115 0.75330345 0.25214579 0.05000000

Zr010 Zr 0.99842827 0.75174471 0.74642188 0.05000000

Zr011 Zr 0.00000000 0.00000000 0.00000000 0.05000000

Zr012 Zr 0.00000000 0.50000000 0.00000000 0.05000000

Zr013 Zr 0.50000000 0.50000000 0.00000000 0.05000000

Zr014 Zr 0.50509033 0.74531488 0.24572475 0.05000000

Zr015 Zr 0.49957596 0.74513414 0.75462700 0.05000000

Zr016 Zr 0.50000000 0.00000000 0.00000000 0.05000000

Zr017 Zr 0.00000000 0.00000000 0.50000000 0.05000000

Zr018 Zr 0.00000000 0.50000000 0.50000000 0.05000000

Zr019 Zr 0.50000000 0.50000000 0.50000000 0.05000000

O0020 O 0.25024073 0.19725370 0.22998817 0.05000000

O0021 O 0.75004536 0.94892336 0.02381026 0.05000000

O0022 O 0.75760444 0.79916967 0.26941359 0.05000000

O0023 O 0.23427546 0.06250565 0.48245624 0.05000000

O0024 O 0.25612586 0.69286895 0.22415670 0.05000000

O0025 O 0.74980742 0.44517383 0.02190608 0.05000000

O0026 O 0.75063305 0.30628310 0.26763628 0.05000000

O0027 O 0.25091879 0.55617109 0.48126881 0.05000000

O0028 O 0.07385613 0.85231238 0.39721959 0.05000000

O0029 O 0.92794607 0.10305786 0.35425260 0.05000000

O0030 O 0.55216383 0.15688922 0.09314705 0.05000000

O0031 O 0.45360421 0.90240573 0.15209418 0.05000000

O0032 O 0.94857532 0.15175398 0.10097247 0.05000000

O0033 O 0.06234435 0.89777901 0.14636922 0.05000000

O0034 O 0.45351277 0.83564323 0.39383851 0.05000000

O0035 O 0.55123068 0.10376502 0.33407037 0.05000000

O0036 O 0.06609799 0.35105125 0.39864067 0.05000000

O0037 O 0.95244273 0.59774839 0.34836225 0.05000000

O0038 O 0.56685587 0.64955445 0.09671847 0.05000000

O0039 O 0.43495865 0.40563789 0.14895161 0.05000000

O0040 O 0.94857728 0.65131044 0.10007895 0.05000000

O0041 O 0.05375410 0.40073697 0.15029303 0.05000000

O0042 O 0.44640213 0.34904644 0.39933660 0.05000000

O0043 O 0.56151972 0.59539835 0.34864376 0.05000000

#End data_Ca_16_Zr_15_O_48_

**Ca_16_Zr_16_O_47_**

data_Ca_16_Zr_16_O_47_

_cell_length_a 11.613816

_cell_length_b 11.240515

_cell_length_c 8.072617

_cell_angle_alpha 90.000000

_cell_angle_beta 90.000000

_cell_angle_gamma 90.000000

_cell_measurement_temperature 0.0

_diffrn_ambient_temperature 0.0

_symmetry_space_group_name_H-M 'Pm '

_symmetry_space_group_number 6

_refine_date '11-12-2016'

_refine_method 'generated from Wien2k code'

_refine_special_details

;

Structure converted from Wien2k struct file, Version 9.1

File Name /home/alay-e-abbas/Desktop/Optimized_Structures_CZO/struct/Ca16Zr16O47/Ca16Zr16O

Title 'CZO_optimized'

;

loop_

_symmetry_equiv_pos_as_xyz

+x,+y,+z

+x,+y,-z

loop_

_atom_site_label

_atom_site_type_symbol

_atom_site_fract_x

_atom_site_fract_y

_atom_site_fract_z

_atom_site_U_iso_or_equiv

Ca001 Ca 0.99828274 0.99776399 0.00000000 0.05000000

Ca002 Ca 0.79886257 0.25158342 0.50000000 0.05000000

Ca003 Ca 0.04936511 0.01185336 0.50000000 0.05000000

Ca004 Ca 0.24966325 0.76261147 0.00000000 0.05000000

Ca005 Ca 0.50022239 0.99998163 0.00000000 0.05000000

Ca006 Ca 0.30204887 0.24933211 0.50000000 0.05000000

Ca007 Ca 0.54984840 0.01346165 0.50000000 0.05000000

Ca008 Ca 0.80040659 0.74955590 0.50000000 0.05000000

Ca009 Ca 0.29961117 0.75073644 0.50000000 0.05000000

Ca010 Ca 0.55006790 0.51307228 0.50000000 0.05000000

Ca011 Ca 0.74932719 0.76336106 0.00000000 0.05000000

Ca012 Ca 0.99945672 0.49672783 0.00000000 0.05000000

Ca013 Ca 0.05042758 0.51499763 0.50000000 0.05000000

Ca014 Ca 0.25316683 0.26348699 0.00000000 0.05000000

Ca015 Ca 0.50005110 0.50004685 0.00000000 0.05000000

Ca016 Ca 0.75452858 0.26164996 0.00000000 0.05000000

Zr017 Zr 0.02482689 0.75574761 0.75067361 0.05000000

Zr018 Zr 0.27455463 0.00650579 0.75118415 0.05000000

Zr019 Zr 0.52483158 0.75682031 0.74970410 0.05000000

Zr020 Zr 0.77500417 0.00626693 0.74978601 0.05000000

Zr021 Zr 0.02525755 0.25796391 0.74722601 0.05000000

Zr022 Zr 0.27529042 0.50630221 0.74978513 0.05000000

Zr023 Zr 0.52605693 0.25659682 0.75064087 0.05000000

Zr024 Zr 0.77469015 0.50677243 0.75044381 0.05000000

O0025 O 0.79432399 0.05786136 0.00000000 0.05000000

O0026 O 0.29734527 0.06054999 0.00000000 0.05000000

O0027 O 0.25320668 0.95462714 0.50000000 0.05000000

O0028 O 0.25409573 0.45180848 0.50000000 0.05000000

O0029 O 0.00457619 0.80987331 0.50000000 0.05000000

O0030 O 0.54639005 0.20244005 0.00000000 0.05000000

O0031 O 0.75482900 0.95241856 0.50000000 0.05000000

O0032 O 0.75299453 0.45366559 0.50000000 0.05000000

O0033 O 0.50337257 0.81092356 0.50000000 0.05000000

O0034 O 0.54511611 0.70332972 0.00000000 0.05000000

O0035 O 0.04595662 0.70165874 0.00000000 0.05000000

O0036 O 0.00039104 0.31391211 0.50000000 0.05000000

O0037 O 0.79558615 0.56092305 0.00000000 0.05000000

O0038 O 0.29580626 0.56007280 0.00000000 0.05000000

O0039 O 0.50509301 0.31008425 0.50000000 0.05000000

O0040 O 0.87502596 0.85506281 0.80698396 0.05000000

O0041 O 0.92592862 0.10535228 0.69547661 0.05000000

O0042 O 0.17394108 0.15900043 0.30438927 0.05000000

O0043 O 0.12515269 0.90718033 0.19252821 0.05000000

O0044 O 0.37449479 0.85553406 0.80658721 0.05000000

O0045 O 0.42444279 0.10610349 0.69262181 0.05000000

O0046 O 0.67532031 0.15762390 0.30769905 0.05000000

O0047 O 0.62456431 0.90796203 0.19374681 0.05000000

O0048 O 0.87505165 0.35637927 0.80534109 0.05000000

O0049 O 0.92450449 0.60550902 0.69223280 0.05000000

O0050 O 0.17474870 0.65707947 0.30757628 0.05000000

O0051 O 0.12579085 0.40604224 0.19226176 0.05000000

O0052 O 0.37610693 0.35587350 0.80663070 0.05000000

O0053 O 0.42477295 0.60576658 0.69230521 0.05000000

O0054 O 0.67455008 0.65856372 0.30725999 0.05000000

O0055 O 0.62496146 0.40812233 0.19285659 0.05000000

#End data_Ca_16_Zr_16_O_47_

**V_2O1_**

data_V2O1

_cell_length_a 5.620258

_cell_length_b 11.613816

_cell_length_c 8.072617

_cell_angle_alpha 90.000000

_cell_angle_beta 90.000000

_cell_angle_gamma 90.000000

_cell_measurement_temperature 0.0

_diffrn_ambient_temperature 0.0

_symmetry_space_group_name_H-M 'Pm '

_symmetry_space_group_number 6

_refine_date '11-12-2016'

_refine_method 'generated from Wien2k code'

_refine_special_details

;

Structure converted from Wien2k struct file, Version 9.1

File Name /home/alay-e-abbas/Desktop/Optimized_Structures_CZO/struct/V2O1/V2O1.struct

Title 'CZO_optimized'

;

loop_

_symmetry_equiv_pos_as_xyz

+x,+y,+z

+x,+y,-z

loop_

_atom_site_label

_atom_site_type_symbol

_atom_site_fract_x

_atom_site_fract_y

_atom_site_fract_z

_atom_site_U_iso_or_equiv

Ca001 Ca 0.01598979 0.99800255 0.00000000 0.05000000

Ca002 Ca 0.49664286 0.79871929 0.50000000 0.05000000

Ca003 Ca 0.97202000 0.04951696 0.50000000 0.05000000

Ca004 Ca 0.47429235 0.25348128 0.00000000 0.05000000

Ca005 Ca 0.99989118 0.50078883 0.00000000 0.05000000

Ca006 Ca 0.49845423 0.30108961 0.50000000 0.05000000

Ca007 Ca 0.97292568 0.54943600 0.50000000 0.05000000

Ca008 Ca 0.47719557 0.75464554 0.00000000 0.05000000

Zr009 Zr 0.48624112 0.02501935 0.74796492 0.05000000

Zr010 Zr 0.98729386 0.27474539 0.75115340 0.05000000

Zr011 Zr 0.48669730 0.52584374 0.75023716 0.05000000

Zr012 Zr 0.98716952 0.77454145 0.74999258 0.05000000

O0013 O 0.88432497 0.79396148 0.00000000 0.05000000

O0014 O 0.87895694 0.29791485 0.00000000 0.05000000

O0015 O 0.09112601 0.25293392 0.50000000 0.05000000

O0016 O 0.37130407 0.00053085 0.50000000 0.05000000

O0017 O 0.59426658 0.54610880 0.00000000 0.05000000

O0018 O 0.09210343 0.75331658 0.50000000 0.05000000

O0019 O 0.37849918 0.50405763 0.50000000 0.05000000

O0020 O 0.28929026 0.87461641 0.80432590 0.05000000

O0021 O 0.78893423 0.92577674 0.69531231 0.05000000

O0022 O 0.68294832 0.17350462 0.30408512 0.05000000

O0023 O 0.18921088 0.12608557 0.19217330 0.05000000

O0024 O 0.28731401 0.37551273 0.80608313 0.05000000

O0025 O 0.78697953 0.42382207 0.69238417 0.05000000

O0026 O 0.68378719 0.67499440 0.30774173 0.05000000

O0027 O 0.18357373 0.62471435 0.19366744 0.05000000

#End data_V2O1

**V_2O2_**

data_V2O2

_cell_length_a 5.620258

_cell_length_b 8.072617

_cell_length_c 11.613816

_cell_angle_alpha 90.000000

_cell_angle_beta 90.000000

_cell_angle_gamma 90.000000

_cell_measurement_temperature 0.0

_diffrn_ambient_temperature 0.0

_symmetry_space_group_name_H-M 'P1 '

_symmetry_space_group_number 1

_refine_date '11-12-2016'

_refine_method 'generated from Wien2k code'

_refine_special_details

;

Structure converted from Wien2k struct file, Version 9.1

File Name /home/alay-e-abbas/Desktop/Optimized_Structures_CZO/struct/V2O2/V2O2.struct

Title 'CZO_optimized'

;

loop_

_symmetry_equiv_pos_as_xyz

+x,+y,+z

loop_

_atom_site_label

_atom_site_type_symbol

_atom_site_fract_x

_atom_site_fract_y

_atom_site_fract_z

_atom_site_U_iso_or_equiv

Ca001 Ca 0.00000000 0.00000000 0.00000000 0.05000000

Ca002 Ca 0.50277149 0.49484310 0.20230862 0.05000000

Ca003 Ca 0.98106813 0.50039538 0.95375264 0.05000000

Ca004 Ca 0.47841331 0.99618873 0.75202395 0.05000000

Ca005 Ca 0.00703724 0.99590051 0.50231758 0.05000000

Ca006 Ca 0.50500055 0.49479038 0.70159125 0.05000000

Ca007 Ca 0.97802011 0.49409720 0.45183952 0.05000000

Ca008 Ca 0.47896476 0.99938948 0.25289344 0.05000000

Zr009 Zr 0.49072212 0.74501777 0.97628004 0.05000000

Zr010 Zr 0.99180302 0.74621844 0.72696031 0.05000000

Zr011 Zr 0.49218688 0.24646028 0.97714794 0.05000000

Zr012 Zr 0.99183198 0.24695721 0.72756203 0.05000000

Zr013 Zr 0.49308233 0.74613665 0.47697988 0.05000000

Zr014 Zr 0.98644346 0.74623190 0.22764406 0.05000000

Zr015 Zr 0.49246804 0.24603662 0.47712807 0.05000000

Zr016 Zr 0.99402023 0.24743313 0.22674655 0.05000000

O0017 O 0.59354323 0.99441739 0.95773578 0.05000000

O0018 O 0.09951506 0.49619708 0.74820271 0.05000000

O0019 O 0.38635649 0.49659630 0.99695319 0.05000000

O0020 O 0.89240281 0.99471014 0.20509525 0.05000000

O0021 O 0.60186866 0.99591959 0.45585396 0.05000000

O0022 O 0.09789905 0.49672057 0.24725317 0.05000000

O0023 O 0.38383934 0.49577926 0.49804749 0.05000000

O0024 O 0.88444664 0.99638894 0.70713532 0.05000000

O0025 O 0.79864351 0.30098427 0.07564578 0.05000000

O0026 O 0.78596872 0.69003228 0.07912731 0.05000000

O0027 O 0.68915829 0.30118095 0.82689141 0.05000000

O0028 O 0.19123387 0.18966538 0.87658283 0.05000000

O0029 O 0.18974987 0.18992396 0.37756082 0.05000000

O0030 O 0.68685516 0.68879335 0.82507860 0.05000000

O0031 O 0.18687894 0.80173027 0.87818941 0.05000000

O0032 O 0.19694566 0.80646006 0.37164498 0.05000000

O0033 O 0.29990097 0.19356328 0.12890889 0.05000000

O0034 O 0.29276825 0.80405876 0.62560423 0.05000000

O0035 O 0.79582535 0.68957752 0.57632277 0.05000000

O0036 O 0.69092542 0.30077583 0.32713831 0.05000000

O0037 O 0.69384672 0.68308554 0.32973057 0.05000000

O0038 O 0.29416493 0.18847795 0.62690758 0.05000000

O0039 O 0.79392352 0.30361440 0.57751046 0.05000000

#End data_V2O2

**V_4O1_**

data_V4O1

_cell_length_a 5.620258

_cell_length_b 5.806908

_cell_length_c 8.072617

_cell_angle_alpha 90.000000

_cell_angle_beta 90.000000

_cell_angle_gamma 90.000000

_cell_measurement_temperature 0.0

_diffrn_ambient_temperature 0.0

_symmetry_space_group_name_H-M 'Pm '

_symmetry_space_group_number 6

_refine_date '11-12-2016'

_refine_method 'generated from Wien2k code'

_refine_special_details

;

Structure converted from Wien2k struct file, Version 9.1

File Name /home/alay-e-abbas/Desktop/Optimized_Structures_CZO/struct/V4O1/V4O1.struct

Title 'CZO_optimized'

;

loop_

_symmetry_equiv_pos_as_xyz

+x,+y,+z

+x,+y,-z

loop_

_atom_site_label

_atom_site_type_symbol

_atom_site_fract_x

_atom_site_fract_y

_atom_site_fract_z

_atom_site_U_iso_or_equiv

Ca001 Ca 0.00379950 0.98302705 0.00000000 0.05000000

Ca002 Ca 0.49873832 0.40257714 0.50000000 0.05000000

Ca003 Ca 0.02278596 0.90055950 0.50000000 0.05000000

Ca004 Ca 0.53857077 0.50179326 0.00000000 0.05000000

Zr005 Zr 0.51387823 0.95131149 0.75103725 0.05000000

Zr006 Zr 0.01201052 0.44852918 0.74830565 0.05000000

O0007 O 0.40994336 0.90821318 0.00000000 0.05000000

O0008 O 0.90015559 0.49834786 0.50000000 0.05000000

O0009 O 0.61660352 0.99549970 0.50000000 0.05000000

O0010 O 0.71481997 0.24908974 0.80700349 0.05000000

O0011 O 0.20863933 0.15323947 0.69559900 0.05000000

O0012 O 0.31339342 0.65067435 0.30548610 0.05000000

O0013 O 0.81290188 0.74962396 0.19695644 0.05000000

#End data_V4O1

**V_4O2_**

data_V4O2

_cell_length_a 5.620258

_cell_length_b 5.806908

_cell_length_c 8.072617

_cell_angle_alpha 90.000000

_cell_angle_beta 90.000000

_cell_angle_gamma 90.000000

_cell_measurement_temperature 0.0

_diffrn_ambient_temperature 0.0

_symmetry_space_group_name_H-M 'P1 '

_symmetry_space_group_number 1

_refine_date '11-12-2016'

_refine_method 'generated from Wien2k code'

_refine_special_details

;

Structure converted from Wien2k struct file, Version 9.1

File Name /home/alay-e-abbas/Desktop/Optimized_Structures_CZO/struct/V4O2/V4O2.struct

Title 'CZO_optimized'

;

loop_

_symmetry_equiv_pos_as_xyz

+x,+y,+z

loop_

_atom_site_label

_atom_site_type_symbol

_atom_site_fract_x

_atom_site_fract_y

_atom_site_fract_z

_atom_site_U_iso_or_equiv

Ca001 Ca 0.99996672 0.00206297 0.00178601 0.05000000

Ca002 Ca 0.50180298 0.40406290 0.50213876 0.05000000

Ca003 Ca 0.02478232 0.90039547 0.49705160 0.05000000

Ca004 Ca 0.52256504 0.49585839 0.00393080 0.05000000

Zr005 Zr 0.50887096 0.94982468 0.75018933 0.05000000

Zr006 Zr 0.01299616 0.44700082 0.74871859 0.05000000

Zr007 Zr 0.51561611 0.95096278 0.25147090 0.05000000

Zr008 Zr 0.01342169 0.45125080 0.24986463 0.05000000

O0009 O 0.41332090 0.90819053 0.99862324 0.05000000

O0010 O 0.90660608 0.49096609 0.50006994 0.05000000

O0011 O 0.61906475 0.99069755 0.50098258 0.05000000

O0012 O 0.11601205 0.41120036 0.99788835 0.05000000

O0013 O 0.71425699 0.24179368 0.80814593 0.05000000

O0014 O 0.20979701 0.14935046 0.68825728 0.05000000

O0015 O 0.31755532 0.65038386 0.30635105 0.05000000

O0016 O 0.81920151 0.75266328 0.19474484 0.05000000

O0017 O 0.30751558 0.65224018 0.69476809 0.05000000

O0018 O 0.21321940 0.15210168 0.30344753 0.05000000

O0019 O 0.71283172 0.25037469 0.19497847 0.05000000

#End data_V4O2

**V_8O1_**

data_V8O1

_cell_length_a 8.072617

_cell_length_b 5.620258

_cell_length_c 5.806908

_cell_angle_alpha 90.000000

_cell_angle_beta 90.000000

_cell_angle_gamma 90.000000

_cell_measurement_temperature 0.0

_diffrn_ambient_temperature 0.0

_symmetry_space_group_name_H-M 'Pmc21 '

_symmetry_space_group_number 26

_refine_date '11-12-2016'

_refine_method 'generated from Wien2k code'

_refine_special_details

;

Structure converted from Wien2k struct file, Version 9.1

File Name /home/alay-e-abbas/Desktop/Optimized_Structures_CZO/struct/V8O1/V8O1.struct

Title 'CZO_optimized'

;

loop_

_symmetry_equiv_pos_as_xyz

+x,+y,+z

-x,-y,+z+1/2

+x,-y,+z+1/2

-x,+y,+z

loop_

_atom_site_label

_atom_site_type_symbol

_atom_site_fract_x

_atom_site_fract_y

_atom_site_fract_z

_atom_site_U_iso_or_equiv

Ca001 Ca 0.00000000 0.21013023 0.48664302 0.05000000

Ca002 Ca 0.50000000 0.74499321 0.90393653 0.05000000

Zr003 Zr 0.74929911 0.74593040 0.45109726 0.05000000

O0004 O 0.50000000 0.85703148 0.49986790 0.05000000

O0005 O 0.80187429 0.95721457 0.74420121 0.05000000

O0006 O 0.69316524 0.44773212 0.65416024 0.05000000

#End data_V8O1

**V_8O2_**

data_V8O2

_cell_length_a 5.620258

_cell_length_b 5.806908

_cell_length_c 8.072617

_cell_angle_alpha 90.000000

_cell_angle_beta 90.000000

_cell_angle_gamma 90.000000

_cell_measurement_temperature 0.0

_diffrn_ambient_temperature 0.0

_symmetry_space_group_name_H-M 'P-1 '

_symmetry_space_group_number 2

_refine_date '11-12-2016'

_refine_method 'generated from Wien2k code'

_refine_special_details

;

Structure converted from Wien2k struct file, Version 9.1

File Name /home/alay-e-abbas/Desktop/Optimized_Structures_CZO/struct/V8O2/V8O2.struct

Title 'CZO_optimized'

;

loop_

_symmetry_equiv_pos_as_xyz

+x,+y,+z

-x,-y,-z

loop_

_atom_site_label

_atom_site_type_symbol

_atom_site_fract_x

_atom_site_fract_y

_atom_site_fract_z

_atom_site_U_iso_or_equiv

Ca001 Ca 0.48833989 0.05371190 0.25662190 0.05000000

Ca002 Ca 0.99445219 0.45754046 0.74853210 0.05000000

Zr003 Zr 0.00000000 0.00000000 0.00000000 0.05000000

Zr004 Zr 0.50000000 0.50000000 0.00000000 0.05000000

Zr005 Zr 0.00000000 0.00000000 0.50000000 0.05000000

Zr006 Zr 0.50000000 0.50000000 0.50000000 0.05000000

O0007 O 0.90103943 0.95897099 0.24844030 0.05000000

O0008 O 0.39709986 0.54130599 0.75201427 0.05000000

O0009 O 0.20456922 0.29381340 0.05639615 0.05000000

O0010 O 0.19603007 0.30204103 0.44594984 0.05000000

O0011 O 0.30707868 0.80300822 0.44805382 0.05000000

#End data_V8O2
